# Micro-Nanoparticle Characterization: Establishing Underpinnings for Proper Identification and Nanotechnology-Enabled Remediation

**DOI:** 10.3390/polym16192837

**Published:** 2024-10-08

**Authors:** Wesley Allen Williams, Shyam Aravamudhan

**Affiliations:** Aravamudhan Lab, Department of Nanoengineering, Joint School of Nanoscience and Nanoengineering, North Carolina Agricultural and Technical State University, Greensboro, NC 27411, USA; saravamu@ncat.edu

**Keywords:** plastics, MPL, NPL, characterization, pretreatment, spectroscopy, microscopy, MPL remediation, LBL

## Abstract

Microplastics (MPLs) and nanoplastics (NPLs) are smaller particles derived from larger plastic material, polymerization, or refuse. In context to environmental health, they are separated into the industrially-created “primary” category or the degradation derivative “secondary” category where the particles exhibit different physiochemical characteristics that attenuate their toxicities. However, some particle types are more well documented in terms of their fate in the environment and potential toxicological effects (secondary) versus their industrial fabrication and chemical characterization (primary). Fourier Transform Infrared Spectroscopy (FTIR/µ-FTIR), Raman/µ-Raman, Proton Nuclear Magnetic Resonance (H-NMR), Curie Point-Gas Chromatography-Mass Spectrometry (CP-gc-MS), Induced Coupled Plasma-Mass Spectrometry (ICP-MS), Nanoparticle Tracking Analysis (NTA), Field Flow Fractionation-Multiple Angle Light Scattering (FFF-MALS), Differential Scanning Calorimetry (DSC), Thermogravimetry (TGA), Differential Mobility Particle [Sizing] (DMPS), Scanning Electron Microscopy (SEM), Transmission Electron Microscopy (TEM), and Scanning Transmission X-ray Microspectroscopy (STXM) are reviewed as part of a suite of characterization methods for physiochemical ascertainment and distinguishment. In addition, Optical-Photothermal Infrared Microspectroscopy (O-PTIR), Z-Stack Confocal Microscopy, Mueller Matrix Polarimetry, and Digital Holography (DH) are touched upon as a suite of cutting-edge modes of characterization. Organizations, like the water treatment or waste management industry, and those in groups that bring awareness to this issue, which are in direct contact with the hydrosphere, can utilize these techniques in order to sense and remediate this plastic polymer pollution. The primary goal of this review paper is to highlight the extent of plastic pollution in the environment as well as introduce its effect on the biodiversity of the planet while underscoring current characterization techniques in this field of research. The secondary goal involves illustrating current and theoretical avenues in which future research needs to address and optimize MPL/NPL remediation, utilizing nanotechnology, before this sleeping giant of a problem awakens.

## 1. Introduction

**Figure 1:**
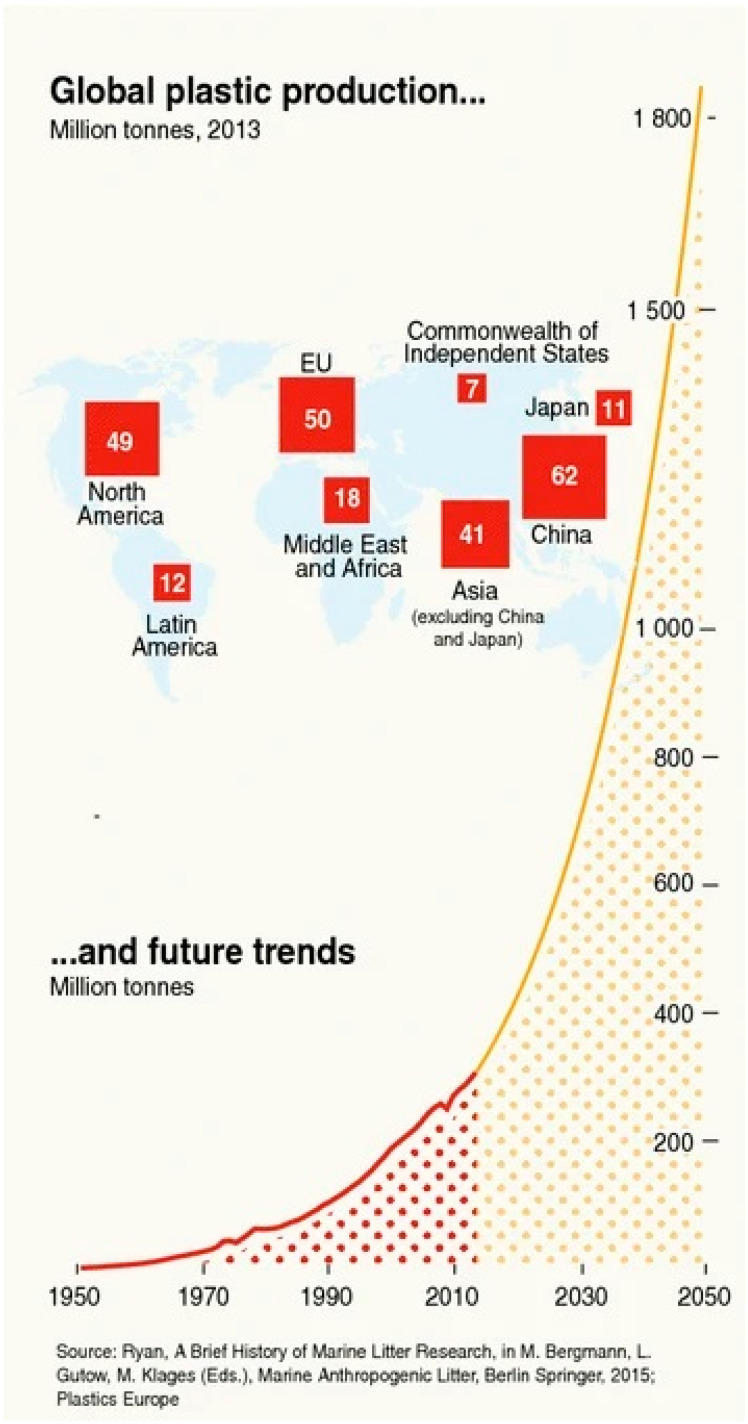
Projection of global plastic production up to the year 2050 and the weights of production estimated by an assortment of continents, countries, unions or regions [2].

Understanding the significance of characterizing MPLs and NPLs begins with addressing the fact that their extent within the environment comes from their induction of larger bulk plastic pollution. Since the 1950s, plastic production has correlated with its induction into the environment, primarily into the hydrosphere [1]. Organization conventions from the UN have sought to address this issue, like the International Convention for the Prevention of Pollution from Ships (MARPOL), by limiting dumping from fishing and other maritime industries. However, it is currently reported that plastic induction primarily starts with wastewater mismanagement from waterways that flow into the greater volume of the hydrosphere. It is estimated [2] that 250,000 tons of the annual induction of plastics are of the MPL type, with one estimate asserting that 150 million tons are presently in the ocean. This is expected to accelerate to a total mass of 1.8 billion tons by the year 2050 (Figure 1).

**Figure 2:**
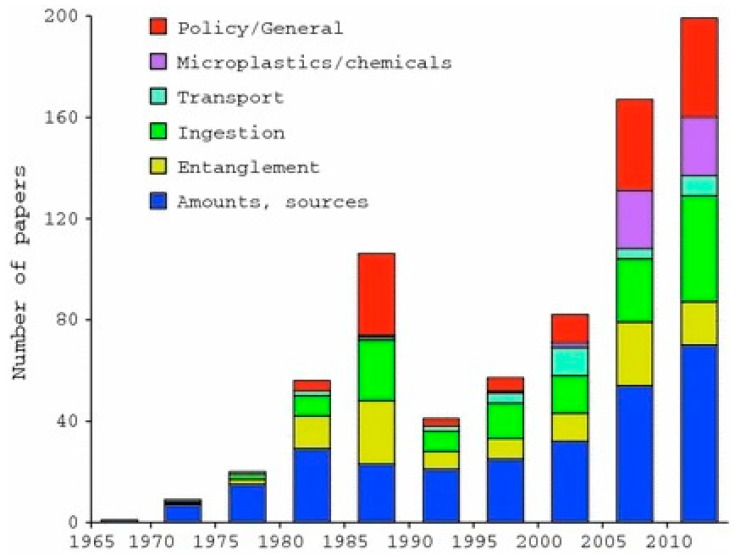
The extent of different areas of plastic research has increased over time in marine science [3].

Consequently, research into plastic pollution began to accelerate around this time [3] (Figure 2) as well, with MPLs and NPLs being elucidated in the late 1900s, negating the notion of plastics’ ability to be hardy from degradation. However, this is more commonly observed in certain environments: highly aerobic or high UV exposed areas. One can infer that free radical formation, scission events, and oxidation can occur for thermoplastics in these conditions, resulting in various degrees of degradation [4,5]. The aforementioned chemical events occur in tandem with physical processes for breakdown, like abrasion, leading to secondary microplastics with higher irregular surface morphology and higher amorphous content. Moreover, these factors often act as an indication of age in MPL characterization [6,7].

An elegant report [8] relayed the theoretical differences and attributes of secondary MPLs and NPLs. They behave differently from their bulk predecessors in that the former (micrometer regime) can form scaffolds and aggregates of biotic or abiotic material and ions/metal cations in the environment to a much higher degree due to a higher surface area to volume ratio (SA:V). To note, many of these cations are heavy transition metals, like Cadmium, which may pose some toxicity [9]. In the latter case (nanometer regime), higher surface energy is often minimized by the adsorption of a heterogenous mixture of material, increasing the chances of aggregation (heteroaggregation). Due to their size, NPLs possess Brownian motion and are subject to random influences from electrostatic forces. This complicates calculation and sampling to ascertain their extent as this phenomenon results in a decreased rate of sedimentation and buoyancy. Lastly, an increase in light interaction (scattering) at this scale contributes to complications with resolving signals from spectroscopic characterization due to more diffuse refraction of light and nanoscale critical dimensions nearing critical DeBroglie wavelengths of notably enhanced plasmons on the surface of these materials compared to bulk material.

Due to this energetically precocious nature, primary (industrially created) and secondary MPLs/NPLs can, unfortunately, introduce themselves in marine ecosystems where uptake, trophic transfer, and human exposure take place. MPLs and NPLs have the preponderance to retain the numerous types of industrial additives found in their native polymer, like flame retardants, pigments, plasticizers, antistatics, antiozonants, vulcanizing agents, curing agents, soaps, surfactants, biocides, metal deactivators, fillers, lubricants, foaming agents, crosslinkers, peptizers and most notably, colorants (Hummel, 2002). A portion of these small molecules are known as persistent organic pollutants (POPs): chemicals that produce a variety of deleterious effects on marine biota and beyond [10]. Moreover, the type of thermoplastics that MPLs and NPLs are made from influences the distribution of additives in the particles themselves and the preponderance of their ability to adsorb extraneous organic molecules, for example, but not limited to antibiotics like ciprofloxacin, sulfamethoxazole, and sulfamethazine; antihypertensives like propranolol; and antidepressants like sertraline and amitriptyline [11,12,13]. More specifically, it is theorized [14] that the rates of adsorption and interaction are influenced by the following theoretical primary intramolecular interactions (note: this is not a completely exhaustive list as there could be more interactions that have yet to be elucidated): polyethylene (PE) having crystallinity dependent interaction with high density polyethylene (HDPE) possessing less opportunity for interaction than low density polyethylene (LDPE), polypropylene (PP) having reduced degrees of freedom due to its bulky isopropyl group (though this may vary based on syndiotactic or isotactic stereochemistry), polystyrene (PS) possessing benzyl structure with interaction from a molecular orbital of delocalized electrons, polyethylene terephthalate (PET) possessing pH dependent interactions but limitations in small molecule size due to electropositive and electronegative centers being in close proximity, and polyvinyl chloride’s (PVC) restrictions with its glassy crystallinity (based on illustrations of the polymer backbone from Ryan [3]).

**Figure 3:**
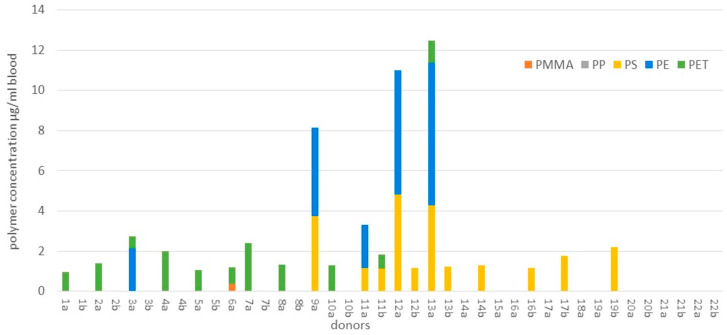
Plastic concentration detected by Curie pyrolysis characterization of blood samples from approximately 80% of 22 healthy volunteers. PE, PS, and PET are among the most abundant plastic types [29].

The effects of these particles and their additives/adsorbents on the environment have mostly been elucidated through experimentation [15,16,17] with engineered MPLs and NPLs. Animals like filter-feeding bivalves, medaka, marine round worms, and sessile organisms have either been shown to uptake and translocate NPLs and MPLs across multiple organ systems, induce oxidative stress and genomic expression shift of receptors of high affinity, or promote invasiveness of species. Furthermore, there are models in the literature that estimate the potential magnification of the POPs found in these particles through trophic transfer, with one [18] possessing reasonable predictability in 30 out of 35 of their species subjects in the Arctic environment which may predicate trophic transfer of MPLs. Higher-order aquatic consumers, like the great white shark, dolphins, the grey seal, and the humpback whale, have been shown to possess a significant amount of MPLs within their gastrointestinal system and their blubber, possibly exerting some damage [19,20,21,22]. Terrestrial consumers, like bears, martens, deer, dogs, and cats (including wild cats), have been shown to possess significant amounts of MPLs inferred from their scat [23,24,25]. Organisms directly involved in human consumption, like produce (i.e., lettuce) and livestock (swine), have been shown to possess significant concentration amounts at a maximum of around eight orders of magnitude of MPLs per gram of produce and the lower hundreds of MPLs per gram of meat [26,27]. Lastly, for human beings, one pathway of exposure, gastrointestinal (GI), is thought [28] to be size-dependent, with the mechanisms in the gut elucidated to be through Peyer’s patches and paracellular uptake of microfold (M) cells. Retention time would vary between the charge nature of these particles, though uptake through dermal contact and pulmonary uptake is theorized as a pathway of inundation potentially complicating kinetics. Lastly, one such paper [29] that came out recently became the first empirical evidence of plastic contamination of PP, PS, PE, and PET in the bloodstream of human beings. Eighty percent of the 22 healthy volunteers who donated their blood to the study possessed, on average, 1.6 µg/mL of plastics, leading to the conjecture that particles in the lower microscale and upper nanoscale range are translocating into the central compartment of human beings (Figure 3). Moreover, it appears that MPLs exhibit a level of lethality, with a report [30] indicating a significant near five-fold increase in the likelihood of exhibiting an end-point cardiovascular event (myocardial infarction/stroke) for patients who possessed MPLs within their atherosclerotic carotid arterial plaques versus patients whose plaques were absent of MPLs.

A caveat detailing the potential trophic transfer of MPLs from the environment to human beings is the confounding exposure though other means. For example, air intake, in tandem with plastic contact, is expected to contribute upwards of 332 ingested MPLs per day [31]. This appears to be a global problem as a subsequent study [32] detailing MPL uptake in human beings from 109 countries across nearly 30 years has found the average concentration to be about 90 mg/capita/day, with southeast Asia predominating. This merely buttresses MPL ubiquity and general uptake. However, multiple modes of uptake must be discussed for comprehensiveness. Pulmonary, dermal, and gastrointestinal uptake will be highlighted as routes of uptake.

In regard to the pulmonary route, it is thought [33] that they primarily deposit in the nasal cavity due to its higher flow rate regardless of spherical or cylindrical (fibrous) nature. However, reports of deposition in the lower respiratory system are present in the literature, with one study [34] in particular reporting the presence of microfiber MPLs resident within embedded human lung tissue. Moreover, there is a higher likelihood of embedment in cancerous tissue. Among the microfiber MPLs characterized were cotton, rayon, and polyester-type MPLs. It was also noted that this abundance compounds with age. Autopsy reports from another study [35] indicated the general presence of polymeric particles smaller than 5.5 µm, of which polyethylene and polypropylene predominated. At the cellular level [36], ROS production is increased from the inflammation pathogenesis of A549 cells in a murine model. Stimulation of p38 and p-NF-κB may induce death of cells in addition to stimulating immune cell recruitment. The plausibility of major inundation is not surprising considering the level of MPL fallout occurring, with one study [37] indicating an MPL abundance of around ~10,000 MPLs/m^2^/day in a typical indoor dormitory.

In regard to the dermal route, one report [38] indicated potential provocation of the skin‘s immune system and disruption of its barrier due to breaching and embedment. Other studies have pointed to additive presence within MPLs as the primary issue, with one report [39] indicating bioaccessible concentrations of polybrominated diphenyl ethers (PBDEs) leeching into the skin with PE contributing to leeching more than PP. Accumulation could eventually lead to liver damage and endocrine disruption. Major sources of these particles [40] include cosmetics and mobile phone cases.

In regard to GI exposure, a few obvious ingestion sources may contribute to MPL induction in the human body, perhaps principally. One report [41] reviewing the presence of MPLs within liquid media in PET bottles determined an overarching range of 4.2 to 5864.1 MPLs/L. In tap water, estimates appeared to be more conservative: 0.2 to 440 MPLs/L. Other sources of plastic packaging could induct MPLs into the body, with one study [42] indicating MPL abundance in common dairy products. As for food sources, common lettuce, *Lactuca*, grown in an urban environment, was found [43] to possess a maximum of 30 MPLs/g of lettuce. Strikingly, one study [44] found an incredible overlap of MPL abundance within seafood, terrestrial meat, and plant-based protein, indicating little impact on food source types except for highly processed meats. This may indicate that meat processing techniques and packaging may be the principal factors of MPL uptake in humans.

## 2. Characterization

### 2.1. Characterization Overview (Introduction)

As mentioned in the previous section, analytical techniques for characterizing MPLs and nanoplastics (NPLs) are an effective methodology for ascertaining their chemical properties and their accumulated changes. Resultant data give remediation and detection methods a stronger basis for their function, efficacy, and safety. The following sections will cover a numerous collection of techniques that identify, quantify, and sample these particles for that very purpose: Fourier-transform infrared spectroscopy (FTIR/µ-FTIR), Raman (or µ-Raman), hydrogen-nuclear magnetic resonance (H-NMR), induced coupled plasma-mass spectrometry (ICP-MS), field flow fractionalization-multiple angle light scattering (FFF-MALS), Curie-point gas chromatography-mass spectrometry (CP-gc-MS), differential mobility particle [Sizing] (DMPS), scanning transmission x-ray microspectroscopy (STXM), fluorescence microscopy, differential scanning calorimetry, (DSC), thermogravimetry (TGA), nanoparticle tracking analysis (NTA), dynamic light scattering (DLS), and turbidimetry. The section will also cover the important differences between the native plastic data to these particles in terms of weathering, oxidation, additive/adsorbent presence, color, adsorption of biotic and abiotic material (eco-corona formation), and so on. Moreover, the section will conclude with newer innovations in sampling methodology for MPLs and NPLs.

### 2.2. Fourier-Transform Infrared Spectroscopy

FTIR detects the changes in the dipolar moment of a bond in a molecule. Some of these bonds possess different magnitudes of frequency based on the size differences of the atoms, their electronegativity, and their interaction with other neighboring molecular bonds (i.e., dipolar induction). In addition, multiple modes or harmonics are not only seen electronically but in the degrees of freedom in which these bonds can bend, stretch, rotate, and so on. As the FTIR interferometer produces a signal, it is the resultant of the different modes that a particle bond can make that result in a peak with relative degrees of broadness when converted to the frequency domain, which typically ranges from 4000 to 1000 inverse centimeters. With good resolution, one can then ascertain the bond type and the degree of the bond. In complex samples, ascertainment becomes increasingly difficult, which, with advanced computational work, might help resolve what specific bonds are in a signal. Instrumental modes like attenuated total reflectance (ATR) can work with dry samples using the resultant reflection of an incident interferometer modified-infrared beam through a crystal. The sample sits on the crystal by which the light reflects immediately off of the sample at the crystal/sample interface into a detector. The detection limit of the FTIR, namely µ-FTIR, is typically 10 µm [45]. This reduces the resolution of particles at or below this limit, thus driving the uncertainty of chemical identification.

**Figure 4:**
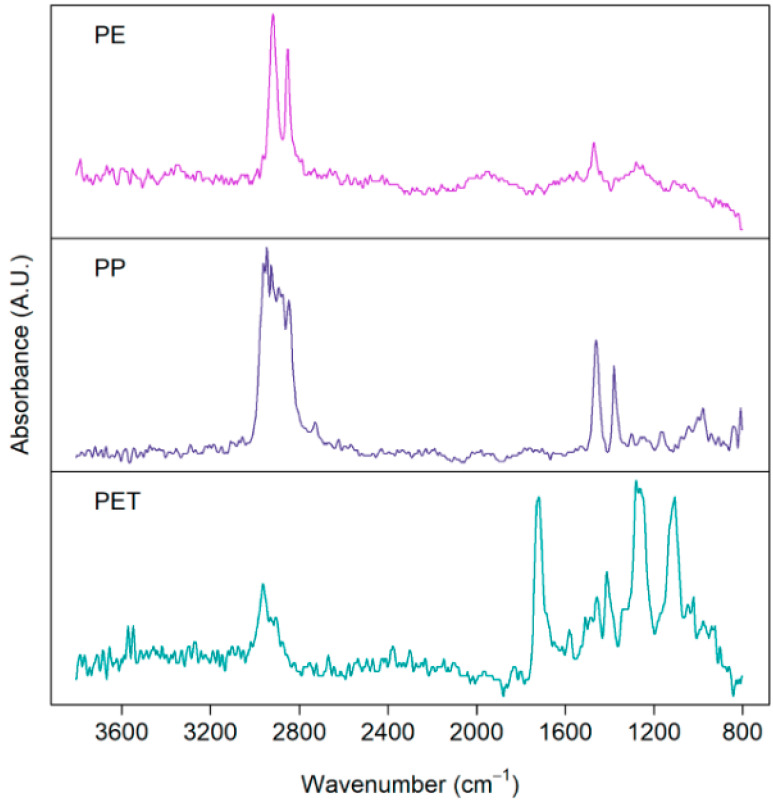
ATR-FTIR spectra of PP/PET/Nylon/PS [46].

In the context of MPLs and NPLs, a library of their native plastic polymers is available in public databases, giving researchers access to compare and score the similarities of the spectra from environmental samples to their bulk native spectra [7]. Specifically, for MPLs and NPLs, we can see (Figure 4) evidence of environmental weathering for these PE, PP, and PET MPLs with considerable noise and characteristic peak broadening in their spectra compared to native spectra. The research group also noted a change in the modality of the spectra of PE MPLs where divergence increases (bimodality of peak) as a function of increased amorphous character in the sample (lowest to highest spectrum). Interestingly, results [46] indicated the degree of intensity of noise obscure characteristic peaks in PET MPLs as a function of increased surface roughness from simulated mechanical abrasion. Moreover, various types of sandpaper with increased degrees of roughness were used as the treatment. Extensive weathering can also be seen here in a similar study [47] where researchers sampled terrestrial environments from the city of Dongguan, in China, resulting in the discovery of weathered cellulose, PE, PP, and PS MPLs from atmospheric deposition similar to a Parisian report [48] they closely replicated. Due to the shape also being in fibrous form, the team concluded that induction was from the textile industry.

In terms of oxidation, prolonged exposure can exhibit degrees of oxidation in MPLs and NPLs, where the introduction of bond stretches attributable to hydroxyl or carbonyl groups begin to form. Carbonyl inclusion of this group is reported [49] in more detail, comparing their intensities between timepoints from 24 to 456 h. Increasing intensity from the inclusion of carbonyl, hydroxyl, and carboxylate functional groups is notable. In addition, in an industrial paper’s example [50], the group induced weathering over an 1138-h period and studied similar effects of oxidation in FTIR on meso/microplastic particles from HDPE, LDPE, PP, and PS. As is expected, the later timeline from 442 to 1138 h possessed the most extensive oxidation with PS showing extensive oxidation in comparison with PP and PE.

### 2.3. Raman Spectroscopy

Raman spectroscopy is governed by the polarizability of atoms’ electronic orbital structure, resulting in a magnitude or shift of EM emission from EM irradiation. This generates a unique spectrum that indicates characteristic bond types of a sampled material. Anti-Stokes, part of inelastic scattering, subtracted from the energy of incident light, gives us precise information on the bond type. This technique possesses remarkable spatial resolution in comparison to FTIR (about 10 to 20-fold higher), benefiting characterization in the MPL and NPL regime [51]. Vibrational modes, similar to FTIR, give rise to a curve around a characteristic wave number that indicates the degrees of freedom the bond structure possesses. Group theory dictates the modes expected from this spectroscopy to be “3N-6” (above linear) or “3N-5” (linear), with N being the number of atoms. This tells us how the bond is moving in context to the adjacent molecular structure under excitation as a simple linear model will result in a lower number of peaks from stretching versus a complex structure (i.e., tetragonal) exhibiting various ways of bending, stretching, rocking, etc. In terms of nanomaterials, forbidden modes are exhibited, which complicates the resolution of the spectrum, especially at the edges of irregularly shaped-nanomaterials. Surface phonon modes, adsorption of the solvent, and surface functionalization from oxidation alter the characteristic wave number and resultant absorbance peaks, also making them less resolute. In terms of the probability of receiving Anti-Stokes scattering for detection, it is primarily dependent on the Maxwell–Boltzmann theory of electron distribution at room temperature, which leads to a greater number of electrons on higher vibrational modes than the ground state which, when irradiated with photons, results in the emission of photons with higher energy as electrons return to ground state producing a negative shift with lower wavelength photons. Generally, the inverse reciprocal of the energy differences of the incident photon wavelength and the Anti-Stokes photon is proportional to and part of the resultant Raman spectrum. In terms of the instrumental parameters, excitation wavelength (lower wavelength lasers producing greater intensity), sample acquisition rates (scans per second), and sampling time help maximize resolution in the spectrum with different trade-offs of time, sample fluorescence, and so on. Optimizing these parameters [51] may be of benefit to researchers who test heterogeneous materials like MPLs and NPLs, perhaps with a Design-of-Experiments (DoE) that can build a preset condition for material or additive/adsorbent nature. Specific modes, like “Raman tweezers”, improve upon this even more so, which will be explained shortly. The limit of detection of Raman, particularly for µ-Raman, is typically 1 µm [52]. This is due to visible light irradiation of the sample as opposed to infrared light, thus lowering the uncertainty of characterization for MPLs within the lower micrometer regime. Compared to µ-FTIR, it is 10 times as resolute.

**Figure 5:**
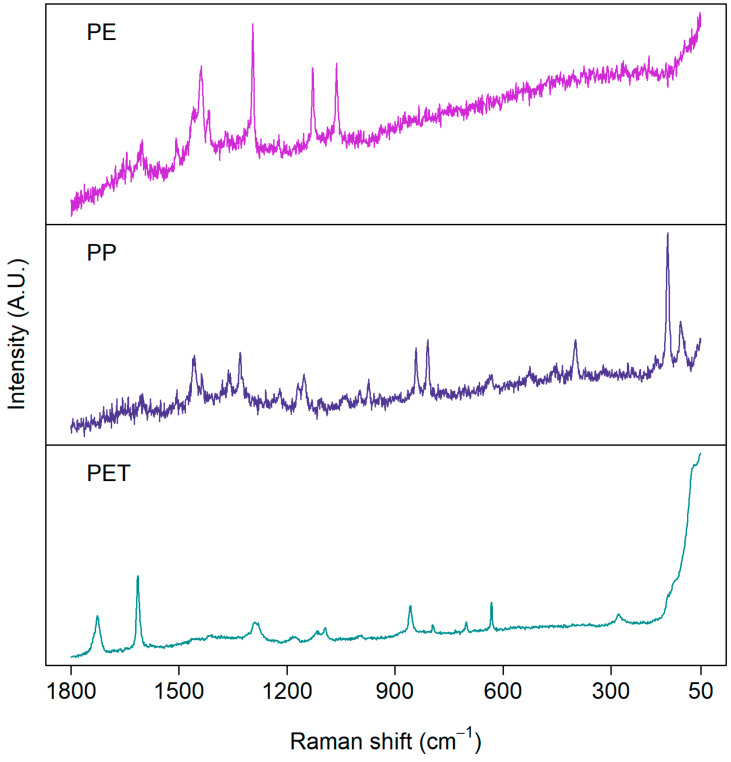
Coastal New Zealand PE/PP/PET plastic particle samples showing uncharacteristic noise [46].

Similarly to FTIR, Raman online spectral databases exist for plastic polymers. Pulled from “PublicSpectra” [53], specifically, are the typical Raman spectra from PE, PP, PS, PET, PVC, and Nylon-6,6. The spectra share some similarities with Raman, where one can find the CH2/CH3 stretches and bends. Single bond C–O, C–C, and phenolic-H is seen in more detail than FTIR, which shows the importance of this technique as a vital complimentary identification technique. A report from New Zealand showcased the significance of weathering via Raman, as seen in [54]. Peak comparison indicates a slight decrease in intensity with a slight increase in uncharacteristic noise. Unfortunately, the article dictates that the size of MPLs measured were from 2 to 5 mm, indicating that they are in the far upper range of MPLs (similarity to bulk is higher, which may not be indicative of effects in the MPL/NPL size regime). This may also explain why the spectra have changed only slightly compared to native spectra. In comparison to the next paper’s example [46], more dramatic changes are seen (Figure 5) where the range of intensity between certain wave numbers is severely diminished, the “width” or uncertainty of their peaks is increased, and the heightened uncharacteristic noise is more severe. This indicates difficulty in resolution, possibly due to physiochemical forces in environmental weathering. Surface roughness, or the proportion of increasing amorphous character, may be to blame based on reports from FTIR with the rationale of it being another interferometry-based spectroscopy method with similar signal processing. In terms of NPLs, a report [55] using the “Raman Tweezers” method sampled six types of MPLs and NPLs: PET, PVC, PP, PE, PMMA, and Nylon-6,6. This method uses the focal point of EM radiation to confine the particles singularly. For MPLs, greater resolution and less noise were exhibited (PET, PVC, PP, and PE); however, for NPLs, significant increases in noise were exhibited in comparison. Interestingly, the mode is able to determine the number of particles linearly, based on the magnitude of the intensity of the characteristic peaks from the samples, as well as distinguish between a particle in the nanoregime versus the microscale regime, which may give valuable insight into investigating the ability for Raman to quantify or approximate the particle concentration of MPLs and NPLs and perhaps other materials in this size regime.

**Figure 6:**
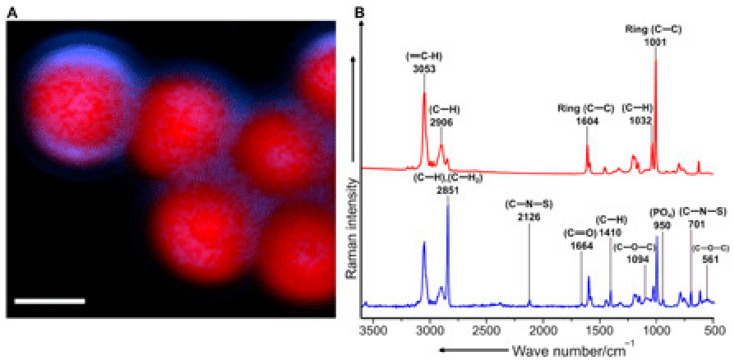
(**A**,**B**) Raman spectrum of engineered PS nanoparticles incubated with environmental water sample forming detectable eco-corona [56].

With respect to biotic sample components, one paper [56] incubated PS NPLs in environmentally-derived water to look for changes in native spectra. Their findings (Figure 6) showed a significant decrease in characteristic peaks from the PS with a heightened increase in functional groups owing to proteins from a biotic “eco-corona”. This underscores the importance of sample pretreatment and the push for characterization techniques that can identify MPLs and NPLs in situ whilst elucidating biotic signals that may be vital for signal processing of MPL and NPL characterization. With respect to color (Portable Raman Microscopy for Identification of Microplastics), polyethylene mesoplastics that were sampled from 200 µm towing net stations were found to have color-dependent noise increase in their spectra which, while being uncharacteristic of MPLs and NPLs, still elucidates the fluorescence effect of pigments.

**Figure 7:**
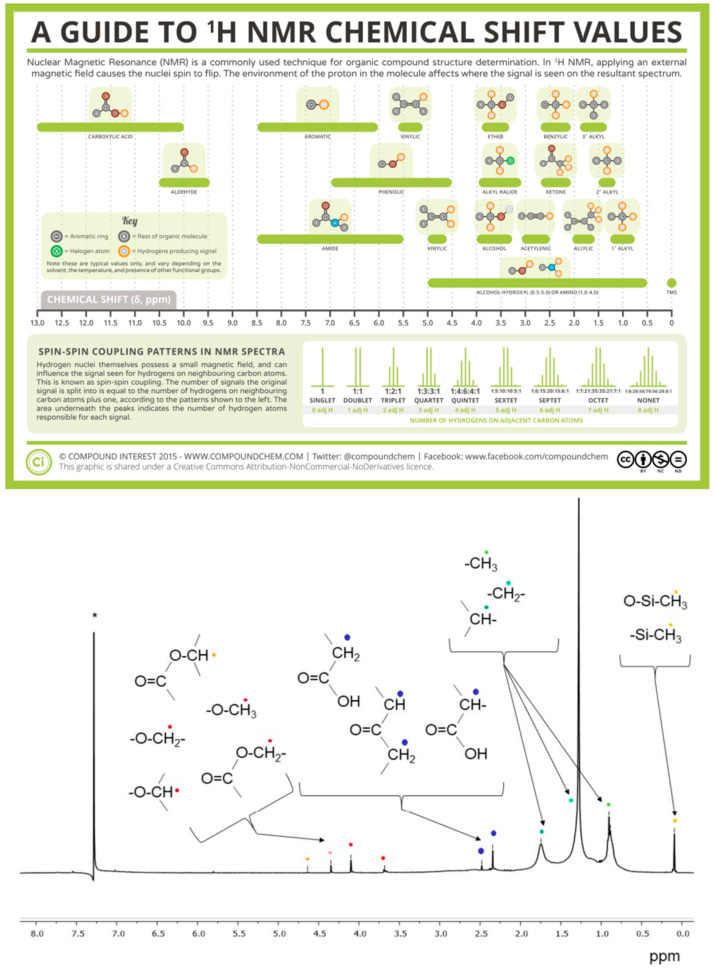
(**Top**) Typical H-NMR shifts for bond type and functional groups. (**Bottom**) Formation of oxidation byproducts detected by H-NMR [58,59]. * singlet at 7.26 ppm from CHCl_3_ traces in CDCl_3_.

In regard to the effect of oxidation seen in Raman, one such research report [57] simulated weathering on PE MPLs which resulted in total occlusion of the prominent CH_2_ peak its native spectra possesses. Furthermore (Figure 7), it was also found that a steady increase in the characteristic peaks of a variety of carbonyl, ether, or ester functional groups was introduced by free radical oxidation.

### 2.4. Proton Nuclear Magnetic Resonance Spectroscopy (H-NMR)

^1^H-NMR is most beneficial for single compound analysis, but its use can extend to polymers and their additives, giving researchers more information into the makeup of their molecular structure. The method detects the induced magnetic alignment of hydrogen nuclei and the resulting magnetic coupling of nearby hydrogen nuclei. The magnetic dipole moments flip its spin direction aligning with an applied magnetic field. From this, we can determine the number of hydrogen atoms on a constituent “branch” of a molecule and determine the distance from the induction of their spin-coupled “neighbors”. The multiplicity of this signal indicates the number of neighbors, including the original center of induction (singlet, doublet, triplet, and so on). This gives analytical chemists a great advantage in reconstructing the sample’s molecular structure. Moreover, there are fewer errors due to proper mapping, focusing, and fluorescence correction being of utmost importance in spectroscopy. The chemical shift is generated by the characteristic relaxation times typically denoting the strength and electronegativity differences of the molecule’s bonds (alkyl hydrogen possessing lower shifts and then carboxylic) (Figure 7: Top) [58]. The limit of detection for ^1^H-NMR varies across the literature, with one report [60] detailing the range around 19–21 μg/mL for PE (D: <300 µm), PET (L: ~500 µm), and PS (D: 500–1000 µm) samples. A subsequent report [61] detailing PS and acrylonitrile butadiene styrene (ABS) detected a mass-based LOD at 2.9 and 0.6 μg, respectively. In a benchtop NMR (500-MHz/80-MHz) [62], LODs for PET (L: ~500 µm|D: 10–20 µm), PVC (D: 13–17 µm), and PS (D: 100–300 µm) were reported to be 1 and 4 μg/mL; 42 and 19 μg/mL; and 9 and 21 μg/mL, respectively.

In terms of MPLs and NPLs, one report [60] was able to detect engineered PS, PET, and LDPE MPLs denoting couplings and shifts present in bulk native spectra. Only slight changes in shifts were recorded, but this may not give enough information into what real environmental samples would look like, though what should be underscored in this paper is that NMR is valuable in the size-independent nature of its characterization. As mentioned earlier, good characterization of MPLs and especially NPLs, needs special modes of operation. Interestingly, one report [59] may help alleviate that need. The report (Figure 7: Bottom) tested the existence of PE and PS MPLs in nature through NMR but, in addition, was able to identify specific oxidated fragments or chains in the molecule. A newer report [63] analyzed MPL off the Italian coast with both ^1^H-NMR and ^13^C NMR (works by exploiting ½ spin of the unpaired neutron in C13). It was determined that the majority of MPLs found were PE (1.12 and ~0.72 ppm) and polydimethylsiloxane (~0.08 ppm) (PDMS) at a limit of quantification of 1 mg/m^3^ of surface water. Concentration was determined to be 0.7–70 mg/m^3^ (PE) and 3.2 mg/m^3^ (PDMS).

### 2.5. Pyrolysis: Curie Point-Gas Chromatography-Mass Spectrometry and Induced Coupled Plasma-Mass Spectrometry

Curie Point Pyrolysis (CP-gc-MS) is an advanced technique that identifies samples with high discrepancy. The sample is induced with high thermal energy up to molecular breakdown, also known as thermochemolysis, via a ferromagnetic radiofrequency conductor. The sample housing is typically made out of a glass capillary with which carrier gas flows over the sample, picking up thermally-degraded products for the measurement of unique retention times which measure the concentration/weight of a substance. The limit of detection appears to vary across the literature, with the Leslie report [29] indicating the following LODs of MPLs from unspiked and spiked human blood blanks: for PMMA, PP, PS, PE, and PET, the limit was 1.6, and 3.7 ng (methylmethacrylate); 16 and 11 ng (2,4-dimethyl-1-heptene); 18/<LOD and 20/8.6 ng (styrene/styrene trimer); 30/52 and 104/101 (1-decene/1-undecene); and 11 and 27 ng (dimethyl terephthalate), respectively. The LOD was set at three standard deviations away from the average value of the blanks for each MPL polymer type. A different report [64] revealed LODs in their QC analysis of “2.31–4.15 μg/g for PS and 3.87–8.20 μg/g for PMMA”. Induced coupled plasma-mass spectrometry works similarly; however, the major difference in the technique is that it is traditionally used for metals. In the report reviewed below [65], users of the technique must deploy metal nanoparticles to their solutions capable of associating with NPL polymers of varying types, thus facilitating number concentration counts. However, both methods, from a sensitivity standpoint, seem comparable in their efficacy aside from their both being pyrolysis techniques [66]. The LOD ICP-MS varies as each report references possesses specific uses or was of calculating their MPL characterization, with one report [64] noting a range from 0.38 to 1.95 μg/g of translocated MPL in plant tissue samples. Another report, detailed below, possesses a completely different metric.

An interesting report [67] from Hudson Bay was able to characterize the presence of MPLs from fish sampled directly from the marine environment. The paper elucidated a 95% recovery efficiency for the treatment of black (spiked Herring) biotic samples before attempting to measure their treatment group from the bay. It was discovered that the Sprat samples possessed a mean concentration of 3, 6, and 7 µg, indicating potent biotic uptake of MPLs and, perhaps, NPLs. A paper using the same technique [29] implemented, previously mentioned before in the Introduction, measured the concentration of plastic particles in the blood of 22 healthy volunteers. Their implementation followed the method of the highly-sensitive “double shot” mode capable of the thermochemolysis of specific groups of plastics rather than all at one instance. A similar report [68] used this technique to identify PE, PVC, PET, and PMMA MPLs within 17 blood samples and found a mean concentration of 1070 ng/mL with PE predominating. In a different context, a paper [64] testing plant tissue, comparing both methods, elucidated the presence of PS and PMMA NPLs, showcasing uptake in every anatomical region of the plant sample over time. Recovery efficiency was comparable for both indicating evidence for a possible alternate avenue in which trophic transfer of NPLs could take place. In terms of improving upon the technique, one report [65] used gold–gelatin core-shell nanoparticles with carboxylic functionalization to increase the sensitivity of ICP-MS down to single particle discrepancy at a lower limit of approximately equal to 800,000 NPL particles per liter. With regard to the effect of environmental matrices formed around MPLs, a striking report [69] was able to determine a higher degree of photoreactivity of PS, PET, and polybutylene adipate-co-terephthalate MPLs entrenched with dissolved organic matter producing a signal attributable to aromatic unsaturation, a first of its kind report indicating higher rates of degradation for particles with eco-corona.

### 2.6. Miscellaneous Identification, Isolation, and Quantification Techniques

This section will cover other techniques due to their limited presence in the literature compared to the aforementioned techniques. These techniques have exciting applications across the board, with some possessing more general applications than others.

**Figure 8:**
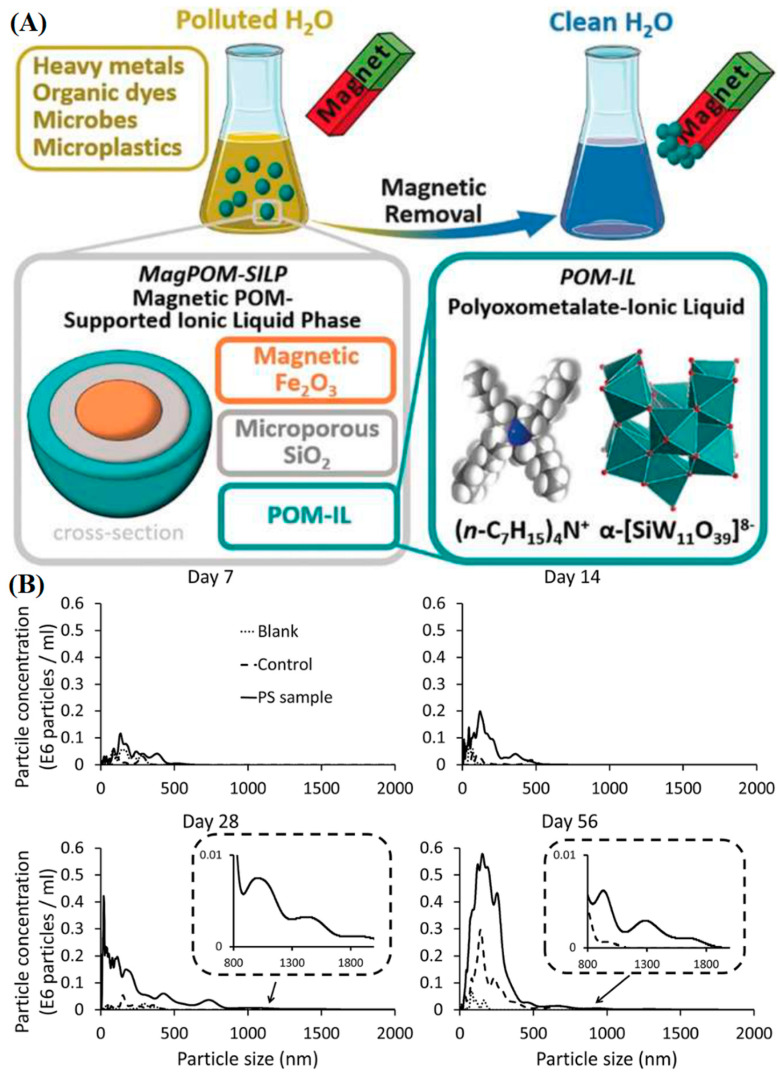
(**A**) Diagram of magPOM-SOLP nanoparticles capable of extracting microplastics and other contaminants through magnetic decantation. (**B**) NTA of evolved MPL and NPL particle concentration from PS irradiated with UV over time [74,83].

In the context of MPLs and NPLs, counting and weighing them is an obvious challenge one must undertake before further experimentation begins. Relatively facile methods, like Dynamic Light Scattering (DLS), can, with adequate standardization, reveal the concentration and size distribution of these particles. Essentially, the measurement is estimated from the spacing measured from scattered light’s constructive or destructive interference into the sensor of the instrument. This gives a sense of the average characteristic dimension of colloids in a dynamic system undergoing Brownian motion [70,71]. The metric is measured under the polydispersity index (PDI), which estimates the heterogeneity of the size distribution of a colloid or suspension of nanoparticles [72]. The detection range varies based on particle weight and apparatus, but it is generally regarded to be 0.005 to 5 µm, limiting its application in the MPL regime [73]. In context to MPL characterization, researchers [74] (Figure 8A) have used this method to quantify the ability of their systems to remediate and remove them. More specifically, the Magnetic Polyoxometalate-Supported Ionic Liquid Phases (magPOM-SILPs) estimated a 90% recovery of MPLs in the system. Moreover, a similar paper [75] measured 98% efficiency with the technique utilizing their crosslinked polyethyleneimine–cellulose fibers “PEI@CE”. Simpler than DLS, turbidimetry utilizes the opacity of a colloid as a measurement of concentration for nanoparticles. The LOD varies based on propriety, but one such technical report [76] states that for the “Lovibond TB Series”, the value is 0.0111 NTU and 0.0135 NTU for white and infrared light, respectively. NTU is the “formazin turbidity standard” unit from the turbidimetry standard solution developed to test proteins by Kingsbury and Clark [77]. One such study [78] implemented a turbidimeter to measure a 98.6% removal efficiency rate for MPLs of varying concentrations based on electrocoagulation from real wastewater samples in a pH range of 4 (and up to 7 in other treatments). A method like this reduces the “workup” necessary for heterogeneous backgrounds. Moreover, two tangential reports measured a 99.4% (4 to 5 orders of magnitude less than the control wastewater sample) and 95% (down from a concentration of 250 ppm) efficiency of clearing MPLs from their systems (passive inorganic metal-based coagulant and electrospun tubular nanofibers) with turbidimetry assessment as well [79,80]. NTA is a similar technique to DLS in that it measure the hydrodynamic diameter of particles in a colloidal suspension although algorithms, software and instrumentation seem to differ (presumably) across companies with different proprietary makeups (Nanoparticle Tracking Analysis NTA|Malvern Panalytical). According to Malvern Panalytical [81], LODs for metals (~10 nm), polymers (~30 nm), and liposomes (~40 nm) differ considerably. Generally [82], the range of particles that can be characterizes is from 30 to 1000 nm with a limit of 10^7^ particles/mL. Nevertheless, it offers itself as a noteworthy tool for characterization. One paper [83] (Figure 8B) implementing this technique wanted to test their hypothesis that NPLs can be formed during the degradation of disposable PS coffee cup lids. The analysis technique, having a boundary of 30 to 2000 nm, found an increase of 0.32 to 1.26 × 10^8^ particles/mL evolved with UV radiation compared to the control (0.04 to 0.41 × 10^8^ particles/mL) at day 14 and day 56.

Field flow fractionalization-multiple angle light scattering (FFF-MALS) is a newer method that quantifies and characterizes particles of many different sizes using electric, magnetic, thermal, centrifugal, or so on to separate particles based on size, electromagnetic properties, etc. Mass transport is the main phenomenon that dictates how the instrument partitions these particles with a channel decorated with pores or helical ribbon-like gaps of different diameters or widths. The application limit of FFF-MALS is attractive in that the various modes, from “flow” to “gravitational”, allow for the detection of particles from 1 nm to 1000 µm, encapsulating the wide size regime of MPLs and NPLs [84]. There is not much literary presence as of yet but it may serve as a valuable technique in the future. [84,85].

Thermogravimetry and Differential Scanning Calorimetry (DSC) are newer techniques in the application of identifying MPLs. Coupled together, they both work to measure the purity of samples based on phase transitions from applied thermal energy. In thermogravimetry [86], the mass of a sample is monitored with respect to heat applied to the sample in a linear fashion. One can assess degradation rates and decomposition mechanisms that elucidate material identity, size of the particulate, and effects of these properties with respect to an oxidative or inert environment. In addition [87], the device can be used to detect phase transitions as well as chemisorption. In DSC [88], heat flow to the sample is measured which elucidates material transitions (e.g., crystallinity changes). Metrics like the “glass transition temperature” can be elucidated. The spectra output possesses components of the glass transition endotherm, the crystallization exotherm, and the fusion endotherm, which determines material behavior or material properties. Concerning limits of detection, the former’s case, for the TGA (PerkinElmer, Waltham, WA, USA) [89], possesses a size limit of about 1 mg with a maximum allowable weight of 1300 mg. The latter’s LOD is about 0.1 mJ [90]; however, in context to the minimum mass of the sample, this will vary based on the amount of heat that can be applied to a particular polymer type (crystallinity/specific heat). One study [91] used these techniques to determine if PE, PP, PVC, polyamide (PA), polyester (PES), PET, and polyurethane (PU) samples could be measured. However, only (weight per volume) concentrations were determined for PE and PP from wastewater samples. Interestingly, it was determined that 34% and 17% of the solid extracted from their sampled consisted of PE, solely illustrating the power of this technique to identify targeted materials in heterogeneous samples. Moreover, a similar report [92] claims to perfect these applications for MPLs by the introduction of multiple heat-up steps that allow more concrete signals to manifest due to the particle‘s semi-crystalline nature. The initial heat-up steps purpose help account for sample impurities increasing SNR. In *Daphnia* [93], another report was able to pair DSC and thermogravimetry techniques to ascertain poly-3-hydroxybutryrate (P3HB)’s concentration spike in the specimens; not only where they were able to detect a notable number concentration via enthalpy, but they were able to notice agglomeration of the MPLs suggesting less likelihood for penetration into the tissues of the specimens presumably lessening the extent of bioaccumulation. In an environmental report [94], DSC was used to determine the presence of LDPE, HDPE, PP, and polycaprolactone (PCL) MPLs within sediment from the Elbe river. Using a standard of eight different polymers, it was estimated that MPLs less than or equal to 100 µm in critical dimension were present in concentrations of 5–44 mg/kg and for particles greater than 100 μm: 0.52 to 1.3 mg/kg.

A similar apparatus using applied thermal energy may be beneficial in measuring the atmospheric content of MPLs and NPLs: Differential Mobility Particle Sizer (DMPS). The instrumentation of DPMS works, in terms of experimental samples, by vaporizing the sample material in a furnace whereby carrier gas flows into cooler regions of the apparatus by which specific temperatures are set to record induced homogenous nucleation. The resultant is fed into a condenser and cooled down with water. Next, the aerosol is combined with compressed air and charged via X-ray irradiation, where the aerosol sample is charged. The analyzer detects the charged particles to determine particle concentration, diameter, and particle type. Using this technique [95], a group was able to measure PET, LDPE, and PP NPLs from about 1 to 100 nm in diameter from a number concentration range that appears to be from 100 particles to 100 million per cubic centimeter in terms of instrumental parameters. Note, that setting temperature, flow rate, and material amount changes the distribution (higher temperature/flow rate/sample mass “pushing” distribution rightward) created with the analyzer, so any comparative replications must take into account the parameters outlined. After standards are made, it may be possible to inject non-spiked atmospheric samples into the machine to compare.

### 2.7. Morphological Characterization

Visual confirmation is an importance aspect of characterizing any material. It is no less important for MPLs and NPLs by which differentiation from other materials, extensive oxidation, surface roughness from abrasion and weathering, cracking, and so on can be seen for their samples. Pairing this with other identification techniques helps shape our expectations and fortifies interpretation. There are only a few notable techniques in literature used to accomplish this task as follows: scanning electron microscopy (SEM), transmission electron microscopy (TEM), atomic force microscopy (AFM), scanning transmission X-ray microspectroscopy (STXM), and Fluorescence Microscopy.

**Figure 9:**
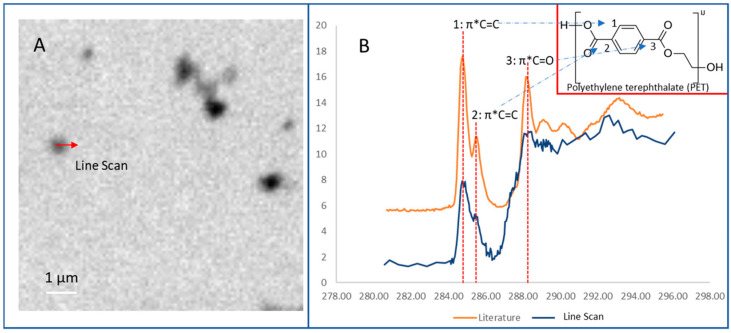
(**A**,**B**) STXM spatial characterization of bonds present within lab-engineered PET NPLs [101].

For SEM, a beam of electrons inundates a sample’s surface and scatters into a detector, resulting in an image of a particle under investigation. In the case of MPLs and NPLs, it gives valuable information into the structure of the particles that indicate aging, oxidation, and polymer type (based on overall morphology). This is typically seen by cracks, holes, groves, and discolorations. It also aids characterization, either pretreated or as is, from their native environments that can be cataloged for comparison with other particles sampled in literature. Paired with previous characterization techniques, it gives a more holistic characterization with adequate sample preparation [6]. The image resolution for the latest SEM is now down to 1 nm [96]. For TEM, the method works similarly in that it bombards the sample with electrons that transmit and detect them in high resolution (atomic), although it is typically used in toxicological models with MPLs and NPLs rather than alone. With TEM, low electrodense materials tend to have a harder time imaging compared with SEM and need to be stained. This can lengthen sample preparation or interfere with obtaining more representative images depending on the staining agent. Also, the staining media is typically made up of heavy metals which can even interfere with the chemical properties of the sample. Conventional TEMs can possess an image resolution of 0.2 nm with below 0.1 nm resolutions possible for the SOTA [97]. For AFM, the probe equipped in the sample can map out a topological-like image that gives great resolution in three dimensions (3D) for MPLs and NPLs; however, the sample needs to be dry firstly [71,98]. The imaging modality, too, possesses incredible spatial resolution at ~1 nm [99]. For STXM, the method scans the sample with focused x-ray beams that generate an image based on the intensity of x-ray flux after transmission. Spectra can be formed in which information on bond type is revealed by a particular coordinate. Lateral resolution is approximately 1 nm with atomic step resolution (~5 nm) [100]. One example [101] employed this technique to verify PET nanoparticles (Figure 9) due to the spectrum possessing carbonyl and pi-star carbon-carbon double bonds. In fluorescence microscopy, a sample is inundated with fluorescent dyes or fluorophores that are associated with thermoplastic particles and provide greater detail of contrast in heterogenous samples. It is unknown to the review as to what the size limit of detection is in regard to spectroscopic characterization. Presumably, it is related to the size range of X-ray photons. Interestingly, a paper [102] employed the usage of Nile Red in an experimentally-derived heterogenous matrix of sand and other environmental samples of LDPE, PP, and expanded polystyrene (EPS) NPLs. Their findings indicated highly distinct structures attributed to the NPLs, with further studies into other thermoplastics with varying results. In addition, this method can distinguish the electronegativity of polymers through solvatochromism [103] (shift of fluorescence). Clearly, optical modes should have a resolution owing to the wavelength of the visible light range of photons.

### 2.8. Isolation and Sample Pretreatment

**Figure 10:**
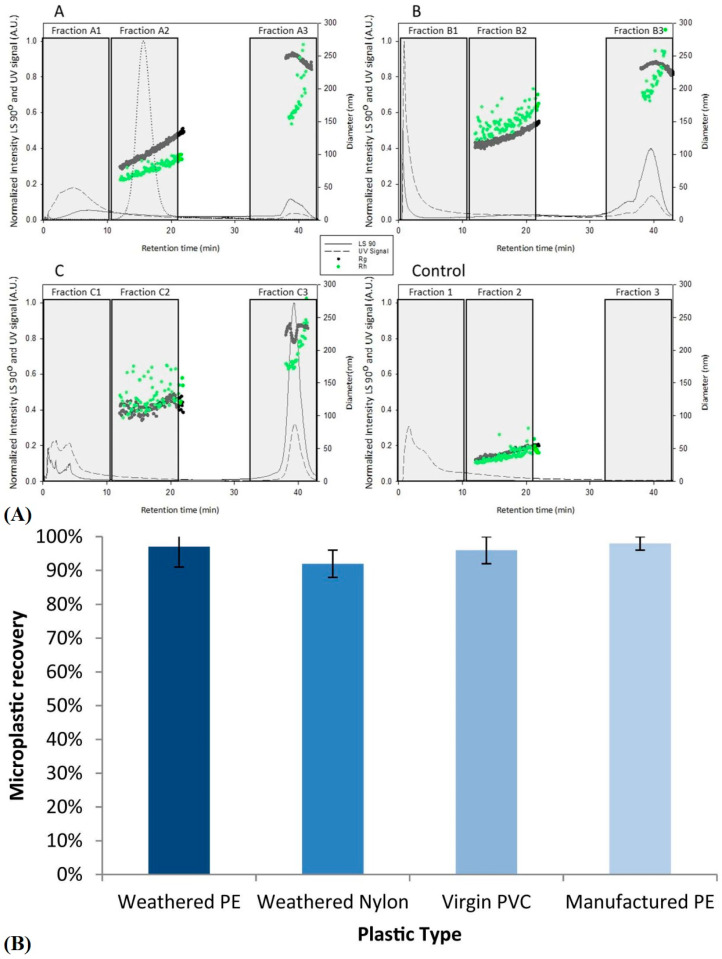
(**A**) A fractogram of three treatment groups and a control of *C. robusta* tissue exposed to 100 nm PS NPLs over time with plus 100 nm results theorized to be aggregation from surface energy minimization. (**B**) The density separation method efficiently using ZnCl_2_ to remove various types of MPLs from spiked estuarine sediment [105,107].

In context to isolation, for terrestrial sources, the issue of losing particles that skew results is not as big of a problem as it is in oceanic or maritime sampling, whereby studies that employ netting to capture microplastics have a lower cutoff with one estimate being about 150 to 300 µm [51]. This confounds the comparison of studies that measure MPL particle sizes at lower regimes in the microscale to the larger scale reports. If oceanic studies are to continue, physical capture by means of the marine biota or sediment should predominate as an isolation technique. For sample isolation in living tissues, the literature uses a wide variety of acids, bases, oxidative agents, and enzymes to purge biotic material that could confound characterization. Interestingly, it [104] was found that the use of nitric acid was too harsh and degraded MPLs from the study, leading to severe underrepresentation. In place, potassium hydroxide and sodium hypochlorite are used, as well as proteinase K. Moreover, as an improvement to these harsh techniques, one study [105] employed the use of papain in tissue samples of *C. robusta* previously bombarded with PS NPLs, resulting in recovery down to a LOD of 1 ng/g of particles from organic tissue. The employment of MALS confirmed the presence of the 100 nm particles increase over time with notable agglomeration evidenced by the detection of diameters above 100 nm (Figure 10A). For isolation techniques of MPLs in abiotic media, membrane filtration cascades whereby sequentially smaller pores are used to help segregate, for example, NPLs from a general solution without clogging. There are other methods, like chromatography, FFF, and electrophoresis, but they have troublesome caveats: higher interaction at the nano regime (size exclusion stationary phase interaction), complex optimization, and functionalization of plastics have to proceed separation before they are influenced by an electric field, respectively [106]. Interestingly, one paper [107] (Figure 10B) illustrates a method that naturally separates heterogenous samples via density by the application of zinc chloride solution that is as efficacious in both engineered and weathered MPLs in artificial incorporation in estuarine sediment. A similar study [108] employed the technique to map out MPL abundance and type in different maritime zones, potentially modeling deposition in finer detail. While the method is cheap and facile, it may not capture particles of all densities. Moreover, particles with an assortment of potential adsorbents and endowed additives may be missed (differing densities/potentially energetically unfavorable), though, as a consolation, there is no isolation protocol that will not leave NPLs and MPLs completely undisturbed. Because of this reality, oleophilic sample media may be of benefit for a more representative capture. One paper [109] took note of the oleophilic properties of MPLs and employed oil in their proof-of-concept to increase the recovery efficiency of sampling protocols suing environmental samples. In addition, a simple ethyl alcohol wash aided in further studying of the sample. In tandem, these techniques would work synergistically as a sort of insurance, though polymers and their constituents have various degrees of oleophilicity (hydrophobicity). Other reviews [110] cite the freeze-drying or dehydrating soils for easier removal, which is less facile, though the primary goal is to employ some solvent that can be used “on-site” during ecological sampling, thus potentially disturbing the samples even less from their native “state”. Further research warrants trials with specific organic solvents, salt solutions, and proteins at various ratios down to specific polymer type which could increase recovery efficiency. There appears to be a greater need to take into account the change in concentration and type-considered abundance of MPLs and NPLs in different environments but a severe gap in the research is how to apply a standard DoE to sample isolation and preparation. Investigating which aspects of protocol may have more weight in recovery based on material type should be of immediate importance to this sub-field.

### 2.9. Characterization Overview (Conclusions)

Table 1 and Table 2 give an overview of the characterization techniques underscored in this review. The techniques possess great benefits towards accelerating the understanding of MPLs and NPLs in every dimension possible, but each possesses its own unique set of issues that makes the need for research proliferation less than ideal (note: the limitations of the table do not address the entirety of the scope of these methods and their capabilities but it addresses a summary of the scope from the review’s extent into researching the topic). The prime limitations appear to be issues regarding extensive sample pretreatment, sample destruction, and signal processing of confounding signals. The former could very well alter the true identification of these particles, the middle issue removes all notions of repeatability in experimentation, and the latter should be investigated in terms of setting new standards for existing MPLs for proper characterization. Computational methods may better resolve and interpret results these results, as well as newer instrumentation in the SOTA.

## 3. SOTA Characterization and Existing/Theoretical Modes of Remediation

### 3.1. SOTA Characterization

An assortment of newer specialty modes of characterization methods, plus new methods, will be expounded upon from the literature. These methods are, currently, the most advanced characterization techniques to identify, quantify, and delineate MPLs from the environment.

#### 3.1.1. Optical-Photothermal Infrared Microspectroscopy (O-PTIR)

O-PTIR [111,112] is a fairly new and unique technique in the literature that relies on the photothermal effect from IR radiation onto a sample. Intensity changes from the effect can be detected by a 532 nm light irradiated onto a sample in tandem for a better resolution. The resolution supersedes traditional IR at an incredible ~450 nm. Compared to traditional IR, the technique is also non-invasive and non-destructive to samples upping reproducibility. One report [113], using this method, detected polydimethylsiloxane (PDMS), PP, PS, PVC, and epoxy resin MPLs within IV delivery system components. The Feret diameter distribution centered around 6–11 µm MPLs, of which, 0.9 to 1.57 MPLs/mL are exuded from the components with the initial condition of no volumetric pump assistance vs. its utilization. An interesting report using this technique, investigating the presence of MPLs within human orchiectomy (testicular removal) samples detected the presence of polyamide, nylon, and ecteola-modified resin with polyamide presenting extensive oxidative damage due to tis broad peak around ~1062 cm^−1^.

#### 3.1.2. Z-Stack Confocal Microscopy

Z-stack confocal microscopy is a simple but effective technique for measuring the volume of particles under investigation. Simply, multiple stacks of images are made in the Z-direction of a particle of interest that is recombined into a three-dimensional (3D) image, of which volumes can be approximated. One report [114], using Nile Red, was able to distinguish MPLs from environmental matrices (non-pristine samples) via Z-stack using threshold-based 3D segmentation. It is a fascinating technique [115] that compares pixels to a threshold value of categorization, meaning the components of each stack’s pixels are differentiated. However, over-segmentation can cause misattribution of eco-corona as MPL and vice versa for under-segmentation.

#### 3.1.3. Mueller Matrix Polarimetry

Mueller Matrix Polarimetry (principle: Mueller matrix spectroscopic ellipsometry [116]) is an advanced technique that generates a wide array of measurable variables to describe MPLs microstructure, size, shape, and characteristic refractive image. The polarimeter relies on the physical phenomenon of electromagnetic polarization. Multiple polarized light illuminations, using a rotating optical element, are irradiated at a sample, which, when refracted off of a particle, give a resultant polarization that can describe its refractive index. However, it is vital to differentiate parallel and perpendicular planes as a reference when reflection occurs (spectroscopic ellipsometry). In the Mueller matrix, excluding the first element, the elements in the first row and the first column indicate diattenuation and polarizance, which detail a reduction in polarization degree and vice versa after interaction with the sample. For the rest of the matrix, one can determine changes in the reflected beam direction in terms of cross-polarization.

One report [117] using this technique was able to differentiate MPLs suspended in seawater in situ. A subsequent older report by the same group indicated differentiation from other microparticles in a marine environment, like microalgae and [118] sand-like particles. Pairing “in-line Gabor holography” with Mueller matrices, in one report [119], appears to detect a volumetric sample of transparent PET microplastics at different transverse planes, enabling better detection (resolution: 1.33 µm). In the traditional microscopy case, orientation angles of particles may inhibit their characterization.

#### 3.1.4. Holographic Imaging

Holographic imaging [120] (specifically, digital holography (DH)) is a measurement technique assessing the refractive index and thickness of a sample under investigation. In principle, a wavefront signal) is propagated, which possesses a phase that is altered under differing dielectric permittivity and light scattering. This gives rise to phase shifts, which are reconstructed.

In context to MPLs, one report [121], utilized DH as a technique needing no pretreatment. Capturing standards (polycarbonate (PC), PET, PVC, PP, PS, and PMMA) involved interference patterns, polarization states, number counts, and morphological characterization all in one apparatus. There is interest [122] in the sub-field to characterize MPLs in this manner via portable high-throughput flow but the application is in its nascence. One promising report from the same group [123] appears to have been able to distinguish PA, PET, and PP MPLs in a prototypical holographic flow-cytometer. Another report [121] indicated the distinguishment of PET, PP, and PMMA MPL sheets, with grayscale value being indicated as a discerning feature. Similarly to the previous report [124], a group utilizing a novel Smart Polarization And Spectroscopic Holography (SPLASH) system combined four polarization states. Moreover, they claim that their technique needs no corroboration from other spectroscopy methods due to their ability to discern particle types from their polarizations. Discernment is also striking against biologically-derived microparticles.

### 3.2. Existing Modes of Remediation

**Figure 11:**
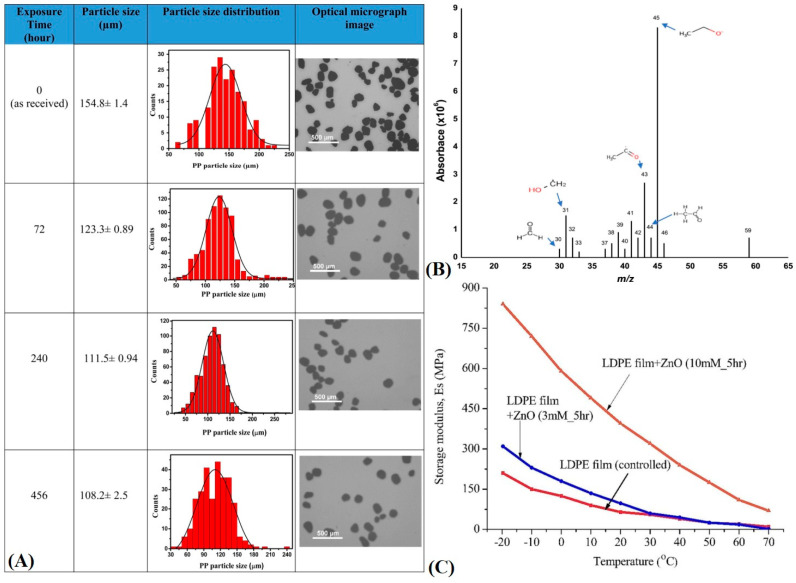
(**A**) Volume reduction from ZnO-NR enhanced-photocatalytic degradation of PP MPLs over time analyzed by DLS. (**B**) GC-MS analysis of byproducts from free radical species produced by photooxidation. (**C**) Elastic moduli decrease with respect to temperature more pronounced in rate and magnitude from higher concentrations of ZnO in comparison to the control, indicating greater physicochemical change in irradiated LDPE due to the presence of oxidation [5,125].

There are multiple avenues of plastic degradation in literature, but the two most relevant pathways are the photolytic and microbial pathways, which could make MPL and NPL remediation feasible in terms of cost and implementation. This is due to, presumably, easier implementation into existing architecture for water remediation and waste management. One report [5] has looked into the advent of zinc oxide nanoparticles capable of reducing the mass of MPLs in a continuous flow system. The PP MPLs flow through glass fiber substrates where they are trapped and subsequently photocatalytically degraded by visible light irradiated-ZnO NRs (Figure 11A,B). GC-MS analysis revealed the formation of ethyl alcohol, organic compounds with hydroxypropyl and acetyl groups, acetylacetone, and acetone: relatively non-toxic byproducts with possible reconstitution (value-added) in chemical engineering processes for common pharmaceutical/industrial compounds. The process is theorized to accelerate the beta-scission mechanism of photooxidation and degradation of PP plastics in a faster time period. Radicals of different sizes (alkoxy radicals) can form that help facilitate scission events but it may be an issue in certain systems not resistant to free radical degradation (Figure 11C). A similar report [125] detailed a similar mechanism of action but found that PE (LDPE), instead, could be degraded in a similar way. SEM revealed morphological cracking and striation formation, an increase in elastic modulus due to extensive oxidation, and the inclusion of functional groups like carbonyls and peroxides in the materials. The primary pathway of degradation was theorized to be through Norrish type reactions of beta-scission as well as superoxide depolymerization. Systems like these could enable waste water treatment plant remediation and ocean venting and filtration, especially due to their ability to work near room temperature.

### 3.3. Theoretical Modes of Remediation via Nanotechnology

#### 3.3.1. Layer-by-Layer (LBL) Nanoparticle Remediator

Firstly, before a process implementing nanotechnology is approved, the proof of concept of the extent of NPLs and MPLs must be established with detection. We then can outline a methodology for creating a nanoparticle platform that can digest MPLs/NPLs with possible integration in water treatment/sewage treatment systems as well as implementation into oceanic events. In order to sense the presence of materials of nanoscale dimensions and concentrations, the advent of a sensing modality capable of being enabled with nanotechnology must be defined. Herein, we will discuss proposed sensing and remediation methods by the review (note: Figures in this section are merely general examples to showcase general methodology or setup).

**Figure 12:**
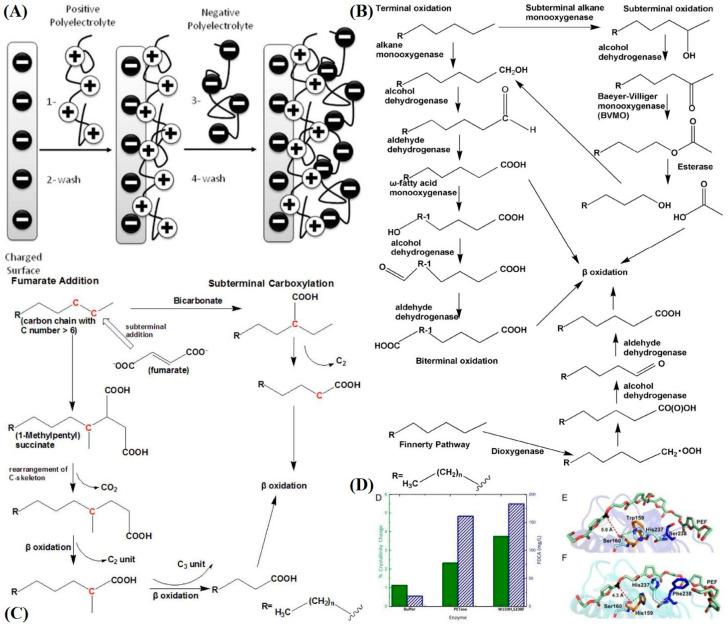
(**A**) Layer-by-layer assembly of polycationic/polyanionic molecules onto a charged surface. (**B**) N-alkane metabolism pathway in *Geobacillus thermodentriticans* for (C > 20) alkanes. (**C**) “Hxd3” strain of *Desulfococcus oleovorans* digestion of n-alkanes via fumarate addition. (**D**) Site-directed mutagenesis of two residues in the PETase enzyme binding pocket increases catalytic capability [126,128,129].

An attractive implementation could be with the use of a biokleptic LBL-directed self-assembled system that ensnares microbial enzymes capable of plastic degradation. A theoretical system like this could possibly preserve their original functionality by increasing their longevity (protection from harsh non-native environments), thus ultimately degrading these harmful particles completely with presumably higher degrees of specificity than the current SOTA. Employment of the use of polycations and polyanions aimed to “sandwich” enzymes (Figure 12A) based on their isoelectric points or overall charge characteristics is contingent upon the specific choice of polymer macromolecules and chemical modification of the system. Moreover, steric and electrostatic interaction must be considered. In addition, adjustment of the porosity of layers over ensnared enzymes to ensure efficient binding opportunity for MPLs/NPLs (or, at least, dangling chains in their amorphous regions) is vital [126]. A study [127] tangential to the LBL nanoparticle system underscored their reasoning as a platform for biokleptic capture of enzymes whilst also bringing to light their preservation of enzymatic activity for their smaller magnetic nanoparticles (their example covalently bonded lactase to the nanoparticles) as follows: 79%, 34%, and 14% for 18 nm, 50 nm, and 200 nm nanoparticle conjugates, respectively. The coefficient of catalysis, k_cat_, decreases with respect to diameter increase, which, according to the paper’s rationale, is due to individual enzyme binding pocket surface interactions with a larger surface area on an individual nanoparticle. As for a LBL system that relies on electrostatic interaction, multiple layers, and different nanoparticle materials reactivity retention will vary. This is fascinating in that the overall higher amount of surface energy for smaller nanoparticles does not disrupt enzymatic activity though this is most likely circumvented by covalent binding, or in this review’s case, by electrostatic interaction. Moreover, emphasis is placed on magnetic NPs due to their enhanced separability from solution.

The choice of enzymes that can be amplified and purified in microbial cultures is an important one in that we want to ensure compatibility with multiple MPL and NPL polymer types as well as their constituents. It is also to ensure they are safe, easily producible, and that their byproducts are safe or separable for value-added manufacturing. Moreover, those byproducts, if environmentally harmful, should be noted and mitigated appropriately. This next subsection will give an overview of potential enzymes of choice that are hypothesized to degrade PE, PP, PVC, PS, PET, and NA6 plastic polymers with some overlap, as well as potential enzymes and antioxidants that can control for free radical/superoxide formation from certain catalytic processes, and finally, control for carbon dioxide production. The final subsection will dive into a quick overview of how these processes could potentially be deployed in a real-world separation process so that the technology is more feasible for end-stage R&D.

#### 3.3.2. Enzyme Selection

There are numerous amounts of plant, fungal, and bacterial enzymes that possess the ability to catalytically degrade plastic polymers, albeit with experimentally challenging process conditions like anaerobic environments, the presence of heavy metal/alkali earth metal cofactors, or mandatory bundling in a particular microbial pathway. Some can be isolated as a sole catalytic enzyme that can bind to a wide array of ligands of interest. A popular one in the literature is “cutinase”, a carboxyl esterase that can hydrolyze the ester bonds of PET polymers with various crystallinity. Lignin enzymes in plants like lignin peroxidase, laccase, and manganese peroxidase, can break down the C–C backbone polymers like PP, PE, PS, and PVC, but they tend to have less enzymatic activity than native lignin due to less redox potential (may be mitigated by environmental oxidation). A report [128] deploying alkane hydroxylase from *E. coli* expansion revealed about a 20% weight reduction of PE with a 10% increase in weight reduction for concomitant alkane monooxygenase, rubredoxin, and rubredoxin reductase. PS, in one example [130], was rapidly degraded to water-soluble byproducts by hydroquinone peroxidase, though it should be noted that this process needs added hydrogen peroxide and tetramethylhydroquinone. A possible pathway yielding recyclable and safe byproducts from plastics was reported (Figure 12B) [128], along with other pathways, via elucidation of a stream-lined isolation of the *Geobacillus thermodentriticans* fatty acid synthesis pathway. This streamline consists of “LndA” (long-chain monooxygenase), alcohol dehydrogenase 1 and 2, and aldehyde dehydrogenase to generate fatty acids of multiple lengths from alkanes. This pathway only works for carbon polymer lengths above 20, but it can be performed without cofactors. Carbon lengths from 2 to 9 can be digested by “a three-component di-iron monooxygenase system that consists of an iron-containing hydroxylase (BMOH), a flavo-iron sulfur-containing NADH-oxidoreductase (BMOR), and a small regulatory component protein (BMOB)” from *Thauera butanivorans*. Fatty acid formation, via the fumarate addition pathway, can occur with at least six carbons seen in a study from the bacterial strain “Hxd3”. After fumarate addition, the product can be used by the bacteria in their beta-oxidation pathway (the caveat is that the exact enzyme is unknown) (Figure 12C). *Thermomyces* (formerly *Humicola*) *insolens* cutinases have been shown to possess complete degradation of PET, although they require high-temperature reaction conditions due to the extremophilic nature of their parent fungi. As for fungal polyester hydrolases, they could overcome the barrier that fungal carboxylesterases possess when encountering crystalline PET MPLs, presumably due to their hydrophobic nature and low SA:V. Interestingly, altered “cutinase” from *Thermobifida fusca* was modified with a disulfide linkage or salt bridge to completely reduce PET of any crystallinity, though the enzyme requires the presence of calcium and magnesium ions. Future steps look towards site-directed mutagenesis of these enzymes. An interesting and somewhat tangential paper (Figure 12D) [129] discovered the increase catalytic activity of one PETase enzyme with two amino acid mutations, allowing PET polymers to sit deeper within the binding complex and coordinate more advantageously with the pi bonds in the benzyl structure of the amino acids in the binding pocket. This ultimately allowed further reduction in the mass of PET than the native enzyme.

**Figure 13:**
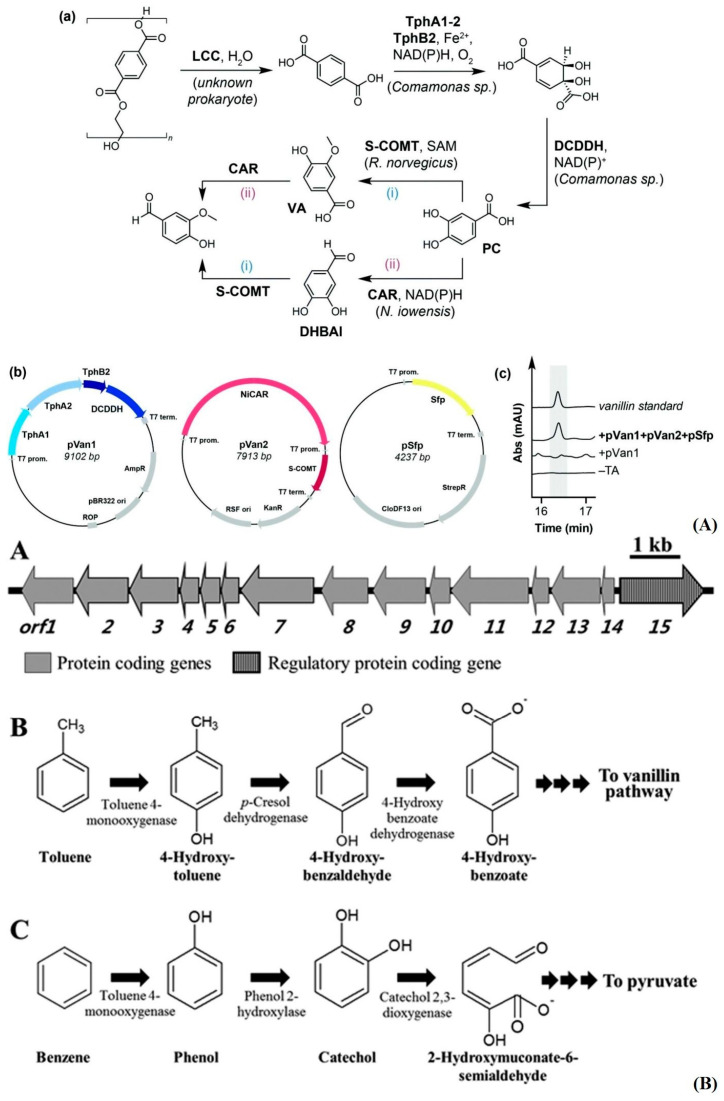
(**A**) Terephthalate modification (monomer of TPS) into vanillin for value-added manufacturing. (**B**) Monomer constituents/enzymes are needed for alternate value-added manufacturing for vanillin and pyruvate metabolic pathways in engineered bacterial strains [131,132].

Taking advantage of bacterial amplification, clustering of enzymes, and their resultant protein amplification could be vital in degradation pathways that circumvent the complexities of isolating individual enzymes or isolating every enzyme responsible in a pathway. For the degradation of PET, one report [131] indicated the use of novel bacterial plasmids in the reduction of terephthalic acid, a final constituent in PET polymers, to vanillin with *E. coli* MG1655 RARE (reduced aromatic aldehyde reduction). Another paper [132] isolated the pathway to pyruvate in (*Pseudoxanthomonas spadix* BD-a59) for benzyl precursors. Saddler’s group fashioned a plasmid for the bacteria by choosing the specific enzymes they wanted to express for their vanillin production from many different species (Figure 13A). Choi’s group performed a bioinformatic study on the genome of their bacteria and found upregulation of peroxidase, monooxygenase, and catalase proteins in the presence of benzyl-containing compounds, which could be possible candidates for PET-compatible enzymes (Figure 13B). Another paper [133] showcasing a sophisticated “multi-omic” bioinformatic study isolated the primary functional enzymes laccase, monooxygenase, dioxygenase, aldehyde dehydrogenase, esterase, and dihydroxy-acid dehydratase in a PVC-degrading strain, “EMBL-1” *Klebsiella variicola*. Moreover, for NA6/NA66, one report characterized nylon digesting enzymes that digest nylon oligomers to single monomer nylon (five carbons between amide bonds). It was found that the activities of 6-aminohexanoate dimer hydrolase A/B and endotype-oligomer hydrolase were responsible [134]. It may take time to delineate which enzymes have an overlap in utility, so minimization of resources occurs, thus maintaining optimal reaction rates and avoidance of steric hindrance for the hypothetical system. Some may have to be partitioned amongst groups of nanoparticles for adequate coverage in various combinations and ratios, while others may work well, concomitantly. Hypothetically speaking, the paper’s focus on MPLs and NPLs should help rapid reaction rates that we ideally want to see in an optimized system (surface area/volume ratio). Whether or not there is application to larger mesoplastics and bulk polymers is out of the scope of this review but merits further investigation.

Unfortunately, the enzymes mentioned above may have the potential to induce off-target effects in an LBL system where free radical scission events could degrade the system faster than ideal. It may be beneficial to incorporate radical scavenging or neutralizing proteins in small fractions that trade off the LBL systems’ enzymatic capabilities for long-term stability. If all else, an environmentally friendly layer of antioxidants may suffice for ease of fabrication and efficacy as a second option, though the caveat is their limited lifetime since enzymes regenerate their reaction center. Applications of superoxides, alkoxy radicals, and peroxides byproducts are generally short-lived since, in the enzymatic sense, they degrade long-chain polymers and alkanes. Unfortunately, this could cause off-target effects (degradation of the LBL scaffold, the system’s components, etc.). Employing antioxidant enzymes such as (Cu/Zn) superoxide dismutase (SOD), catalase (hydrogen donor needed), and glutathione peroxidase (selenium) could potentially help. Many of these enzymes are already expressed in vital plastic polymer degradation pathways and are found resident within human metabolic pathways as well [135]. As for alkoxy radicals, molecular antioxidants like flavonoids may be able to suppress their generation in the system [136]. Laccases, or benzenediol oxygen reductases (copper), may be able to scavenge for a wide variety of phenolic or non-phenolic substances as well as their free radical counterparts. This is vital in the sense that it could neutralize/degrade off-target radicals owing to environmental organic pollutants or POPs within MPLs and NPLs [137]. As an aside to other potential byproducts, if there is the use of short-chain monooxygenases in a series of steps (plasmid gene cluster), for example, as detailed in Ji’s research group, it can produce carbon dioxide, which may make the system inefficient if the gas is produced in copious quantities. This must be mitigated, sequestered, or at least proven as minimally impactful to global carbon dioxide output. Clearly, this will depend on which enzymatic pathways and individual pathways will be in use, but if needed, one such use of metal-dependent formate dehydrogenase may be able to sequester carbon dioxide safely [138].

#### 3.3.3. Processing Implementation in Wastewater Management

In terms of the feasibility of implementing nanotechnology into existing civil engineering architecture, we must take careful consideration into the already existing architecture in wastewater management, waste management, and oceanic clean up. For example, when considering the separation processes in wastewater management, we will have to invent an apparatus or separation that can safely segregate the enzymatic processes of the nanotechnology, the byproducts, and unreacted MPL/NPL reagents before the efflux goes into the environment. This has to be compatible with existing architecture so as to not lengthen the process of its implementation. A purified inflow would fill a hypothetical reactor where MPL/NPL separation and reaction can take place. In some cases, the first concept for detection and separation in the review could help in the process of particles in a separate vessel by elution that can dislodge the captured particles and concentrate them (perhaps by a high salinity solution). In the main reactor, the magnetic LBL nanoparticle system would be separated from the solution, leaving only the byproducts. Mass-separating agents could segregate byproducts that could not be separated by phase. They can then be recycled and used elsewhere in different industries (or discarded if toxic). As for the safer byproducts, they can be incorporated into the efflux from the final clarification process. For errant magnetic NPs, separators between different compartments could magnetically immobilize them before they move on if the initial semipermeable membrane does not suffice (should only allow molecular byproducts to filter through). Connecting pipelines would recycle escaped nanoparticles back, but the magnetic field applied to the main reactor should prevent this last resort. Unreacted MPLs and NPLs could be sequestered by a semipermeable membrane or by simple recycling through the same reaction vessel until desirably safe concentrations are achieved. Taps would be placed at critical areas to monitor conditions via concentration studies and pressure monitors so that membranes are not disrupted by too much hydrodynamic force [139].

## 4. Discussion and Conclusions

Ascertaining the exact composition of MPLs and NPLs is a complex and arduous process. Multiple characterization techniques, computational work, comparison with existing databases and literature as well as replication of protocols are needed to accurately interpret the samples we receive. The heterogeneity and extensive physiochemical changes that occur to these particles in nature, as well as the passive adsorption of confounding and toxic chemicals, complicate the research. An important note must be made: MPLs and NPLs from composite polymers and copolymers were not included in this study. Many of the examples we reviewed investigated the pristine and weathered conditions for mono-polymers. We presume these materials should possess principal wavenumbers owing to 2 or more polymers present assuming ratios are generally equal and there are no larger effects of one polymer’s presentation over the other. As for remediation, various combinations of techniques may have to be deployed to take into account the total nature of these particles.

Newer technologies that can assess theses toxic particles before sensing and remediation is much needed. Nevertheless, if considerable effort is put into standardizing these processes, it will help establish and quicken the proofs-of-concept needed to engineer devices that can differentiate and safely remediate these particles. Even here, form (heterogeneity ascertainment) dictates function (remediation with high specificity). Our industrial society is inundated with harmful toxicants and pollutants of all different types from our own activities as humans. With predicted devastating effects, from a toxicological standpoint, it is paramount to divert attention and resources towards solving this litany of issues if we do not further wish to destroy progress as a species and ourselves. Referring back to Leslie’s and Marfella’s reports, plastic particles are circulating within many human beings, causing damage. These reports are just the beginning of elucidating the toxicology of these particles. There is no telling what how far these impacts extend on the biosphere as a whole but, one comforting fact is for certain: multitudes of research groups across the globe in this seemingly small area of research are working towards a concerted solution and need the support of those involved and those privy to this field.

## Figures and Tables

**Table 1:** Overview of characterization modes. Application towards MPLs and/or NPLs, sample preparation type, and tradeoffs are summarized (Part 1 of 2).

Method	Category	Particle Size Regime	Sample Preparation	Advantages	Disadvantages
FTIR	Identification	MPLs	Dry	Adequate resolution and well-reported method. Identifies polymers, additives, and adsorbents. Spectral libraries available. Resolution: ~10 µm [32]	Sensitive to confounding chemical noise from additives/adsorbents.
Raman	Identification/Quantification	MPLs/NPLs	Dry (Wet: Raman Tweezers)	Higher spatial resolution, well-reported method. Identifies polymers, additives, and adsorbents. Spectral libraries available. Resolution: ~1 nm [39]	Sensitive to confounding chemical noise from additives/adsorbents. Fluorescence from the material can be an issue.
H-NMR	Identification	MPLs	Wet	Possible confirmatory technique for structural analysis. Identifies the structure of oxidized species. LOD: 1 µg/mL/6 µg [46,47,48]	Extensive sample preparation.
Pyrolysis	Identification/Quantification	MPLs/NPLs	Wet	LoD is low for the concentration and size of particles. Great application for biological samples. LOD: 1.6/2.31 µg/g [29,50]	Sample destruction. Extensive sample preparation.
FFF-MALS	Identification/Quantification	MPLs/NPLs	Wet	Various size regimes can be studied based on applied mode (field/pore shape nature and size 0.38 µg/g) [50].	Not well studied. Largely proprietary.
Counting/Weighing	Quantification	MPLs (counting)/MPLs and NPL weighing	Dry	Mainly benefits larger MPLs and mesoplastics.	NPL and lower MPL regime more challenging to weigh/count.

**Table 2:** Overview of characterization modes. Application towards MPLs and/or NPLs, sample preparation type, and tradeoffs are summarized (Part 2 of 2).

Method	Category	Particle Size Regime	Sample Preparation	Advantages	Disadvantages
DLS	Quantification	MPLs/NPLs	Wet (colloid)	Gives the average diameter in addition to the concentration of MPLs and NPLs. LOD: 0.005 µm [56]	Engineered MPL and NPL colloids with adequate serial dilutions for calibration needed. Samples tested must be within range of calibration.
Turbidimetry	Quantification	MPLs	Wet/Dry	Facile: Measures opacity. LOD: 0.0011 NTU [61]	Turbidimetry is not as common as DLS in literature for nanoparticles.
AFM/SEM/TEM	Identification	MPLs/NPLs	Dry	Elucidates morphological nature of particles. Resolution: 1/0.1/1 nm [74,75,77]	AFM is not well-studied for MPLs.
STXM	Identification	MPLs/NPLs/Microfibrils	Dry	Offers spatial determination of material type present in a given sample. Resolution: 1 nm (Step size: 5 mm) [78]	Extensive preparation for particle type and size. Grid needed for centrifugal capture.
NTA	Quantification	MPLs/NPLs (by particle conc.)	Wet	Fairly facile and quick method for particle concentration determination. Resolution 10 nm [64]	Limited to specific ranges of particle concentrations.
TGA	Identification	MPLs (heterogeneous sample may contain NPLs/Mesoplastic, etc.)	Dry	Concentration of PE/PP is determined in a heterogeneous sample. LOD: 1 mg [69]	No clear determination of MPL and NPL presence (general plastic concentration).
DSC	Identification	MPLs (specific extraction for higher regime MPLs)	Dry	Heating steps improve SNR. Resolution: 0.1 mJ [70]	Sample not heterogenous.

## Data Availability

Not applicable.

## References

[1] Bergmann M., Gutow L., Klages M. (2015). Marine Anthropogenic Litter.

[2] Gallo F., Fossi C., Weber R., Santillo D., Sousa J., Ingram I., Nadal A., Romano D. (2018). Marine litter plastics and microplastics and their toxic chemicals components: The need for urgent preventive measures. Environ. Sci. Eur..

[3] Ryan P.G. (2015). A Brief History of Marine Litter Research. Marine Anthropogenic Litter.

[4] Chamas A., Moon H., Zheng J., Qiu Y., Tabassum T., Jang J.H., Abu-Omar M., Scott S.L., Suh S. (2020). Degradation Rates of Plastics in the Environment. ACS Sustain. Chem. Eng..

[5] Uheida A., Mejía H.G., Abdel-Rehim M., Hamd W., Dutta J. (2021). Visible light photocatalytic degradation of polypropylene microplastics in a continuous water flow system. J. Hazard. Mater..

[6] Corcoran P.L., Biesinger M.C., Grifi M. (2009). Plastics and beaches: A degrading relationship. Mar. Pollut. Bull..

[7] Mecozzi M., Pietroletti M., Monakhova Y.B. (2016). FTIR spectroscopy supported by statistical techniques for the structural characterization of plastic debris in the marine environment: Application to monitoring studies. Mar. Pollut. Bull..

[8] ter Halle A., Ghiglione J.F. (2021). Nanoplastics: A Complex, Polluting Terra Incognita. Environ. Sci. Technol..

[9] Ren Z., Gui X., Xu X., Zhao L., Qiu H., Wang X., Cao X. (2022). Weathering of microplastics and their enhancement on the retention of cadmium in coastal soil saturated with seawater. J. Hazard. Mater..

[10] Alharbi O.M.L., Basheer A.A., Khattab R.A., Ali I. (2018). Health and environmental effects of persistent organic pollutants. J. Mol. Liq..

[11] Zhao M., Huang L., Babu Arulmani S.R., Yan J., Wu L., Wu T., Zhang H., Xiao T. (2022). Adsorption of Different Pollutants by Using Microplastic with Different Influencing Factors and Mechanisms in Wastewater: A Review. Nanomaterials.

[12] Atugoda T., Vithanage M., Wijesekara H., Bolan N., Sarmah A.K., Bank M.S., You S., Ok Y.S. (2021). Interactions between microplastics, pharmaceuticals and personal care products: Implications for vector transport. Environ. Int..

[13] Ye Z., Mai T., Cheng Y., Zhang X., Liu Z., Zhang Z., Li Y. (2023). Neurotoxicity of microplastics: A CiteSpace-based review and emerging trends study. Environ. Monit. Assess..

[14] Agboola O.D., Benson N.U. (2021). Physisorption and Chemisorption Mechanisms Influencing Micro (Nano) Plastics-Organic Chemical Contaminants Interactions: A Review. Front. Environ. Sci..

[15] Koelmans A.A., Besseling E., Shim W.J., Koelmans A.A., Besseling E., Besseling E., Shim W.J. (2015). Nanoplastics in the Aquatic Environment. Critical Review. Marine Anthropogenic Litter.

[16] Shao H., Han Z., Krasteva N., Wang D. (2019). Identification of signaling cascade in the insulin signaling pathway in response to nanopolystyrene particles. Nanotoxicology.

[17] Thushari G.G.N., Senevirathna J.D.M. (2020). Plastic pollution in the marine environment. Heliyon.

[18] deBruyn A.M.H., Gobas F.A.P.C. (2006). A Bioenergetic Biomagnification Model for the Animal Kingdom. Environ. Sci. Technol..

[19] Lu H.-C., Smith J.L., Ziajahromi S., Leusch F.D.L. (2024). Microplastics and other anthropogenic fibres in large apex shark species: Abundance, characteristics, and recommendations for future research. Chemosphere.

[20] Sá S., Torres-Pereira A., Ferreira M., Monteiro S.S., Fradoca R., Sequeira M., Vingada J., Eira C. (2023). Microplastics in Cetaceans Stranded on the Portuguese Coast. Animals.

[21] Hernandez-Milian G., Lusher A., MacGabban S., Rogan E. (2019). Microplastics in grey seal (Halichoerus grypus) intestines: Are they associated with parasite aggregations?. Mar. Pollut. Bull..

[22] Garthwaite J. “How Much Microplastic Do Whales Eat? Up to 10 Million Pieces per Day, Stanford Research Finds,” Stanford Report. https://news.stanford.edu/stories/2022/11/whales-eat-colossal-amounts-microplastics#:~:text=Humpbackwhalessubsistingprimarilyon,millionmicroplasticpiecesperday.

[23] Microplastics Found in Feces and Natural Habitat of Taiwan’s Protected Species. Greenpeace East Asia. https://www.greenpeace.org/eastasia/press/7525/microplastics-found-in-feces-and-natural-habitat-of-taiwans-protected-species/.

[24] Zhang J., Wang L., Kannan K. (2019). Polyethylene Terephthalate and Polycarbonate Microplastics in Pet Food and Feces from the United States. Environ. Sci. Technol..

[25] Ratnayaka A.A.W., Serieys L.E.K., Hangawatte T.A., Leung L.K.P., Fisher D.O. (2023). Plastic ingestion by fishing cats suggests trophic transfer in urban wetlands. Environ. Pollut..

[26] Oliveri Conti G., Ferrante M., Banni M., Favara C., Nicolosi I., Cristaldi A., Fiore M., Zuccarello P. (2020). Micro- and nano-plastics in edible fruit and vegetables. The first diet risks assessment for the general population. Environ. Res..

[27] Li H., Yang Z., Jiang F., Li L., Li Y., Zhang M., Qi Z., Ma R., Zhang Y., Fang J. (2023). Detection of microplastics in domestic and fetal pigs’ lung tissue in natural environment: A preliminary study. Environ. Res..

[28] Galloway T.S. (2015). Micro- and Nano-plastics and Human Health. Marine Anthropogenic Litter.

[29] Leslie H.A., van Velzen M.J.M., Brandsma S.H., Vethaak A.D., Garcia-Vallejo J.J., Lamoree M.H. (2022). Discovery and quantification of plastic particle pollution in human blood. Environ. Int..

[30] Marfella R., Prattichizzo F., Sardu C., Fulgenzi G., Graciotti L., Spadoni T., D’Onofrio N., Scisciola L., La Grotta R., Frigé C. (2024). Microplastics and Nanoplastics in Atheromas and Cardiovascular Events. N. Engl. J. Med..

[31] Jadhav E.B., Sankhla M.S., Bhat R.A., Bhagat D.S. (2021). Microplastics from food packaging: An overview of human consumption, health threats, and alternative solutions. Environ. Nanotechnol. Monit. Manag..

[32] Zhao X., You F. (2024). Microplastic Human Dietary Uptake from 1990 to 2018 Grew across 109 Major Developing and Industrialized Countries but Can Be Halved by Plastic Debris Removal. Environ. Sci. Technol..

[33] Islam M.S., Rahman M.M., Larpruenrudee P., Arsalanloo A., Beni H.M., Islam M.A., Gu Y., Sauret E. (2023). How microplastics are transported and deposited in realistic upper airways?. Phys. Fluids.

[34] Chen Q., Gao J., Yu H., Su H., Yang Y., Cao Y., Zhang Q., Ren Y., Hollert H., Shi H. (2022). An emerging role of microplastics in the etiology of lung ground glass nodules. Environ. Sci. Eur..

[35] Amato-Lourenço L.F., Carvalho-Oliveira R., Júnior G.R., dos Santos Galvão L., Ando R.A., Mauad T. (2021). Presence of airborne microplastics in human lung tissue. J. Hazard. Mater..

[36] Woo J.-H., Seo H.J., Lee J.-Y., Lee I., Jeon K., Kim B., Lee K. (2023). Polypropylene nanoplastic exposure leads to lung inflammation through p38-mediated NF-κB pathway due to mitochondrial damage. Part. Fibre Toxicol..

[37] Zhang Q., Zhao Y., Du F., Cai H., Wang G., Shi H. (2020). Microplastic Fallout in Different Indoor Environments. Environ. Sci. Technol..

[38] Aristizabal M., Jiménez-Orrego K.V., Caicedo-León M.D., Páez-Cárdenas L.S., Castellanos-García I., Villalba-Moreno D.L., Ramírez-Zuluaga L.V., Hsu J.T.S., Jaller J., Gold M. (2024). Microplastics in dermatology: Potential effects on skin homeostasis. J. Cosmet. Dermatol..

[39] Abafe O.A., Harrad S., Abdallah M.A.-E. (2023). Novel Insights into the Dermal Bioaccessibility and Human Exposure to Brominated Flame Retardant Additives in Microplastics. Environ. Sci. Technol..

[40] Boccia P., Mondellini S., Mauro S., Zanellato M., Parolini M., Sturchio E. (2024). Potential Effects of Environmental and Occupational Exposure to Microplastics: An Overview of Air Contamination. Toxics.

[41] Gambino I., Bagordo F., Grassi T., Panico A., De Donno A. (2022). Occurrence of Microplastics in Tap and Bottled Water: Current Knowledge. Int. J. Environ. Res. Public Heal..

[42] Kaseke T., Lujic T., Cirkovic Velickovic T. (2023). Nano- and Microplastics Migration from Plastic Food Packaging into Dairy Products: Impact on Nutrient Digestion, Absorption, and Metabolism. Foods.

[43] Canha N., Jafarova M., Grifoni L., Gamelas C.A., Alves L.C., Almeida S.M., Loppi S. (2023). Microplastic contamination of lettuces grown in urban vegetable gardens in Lisbon (Portugal). Sci. Rep..

[44] Milne M.H., De Frond H., Rochman C.M., Mallos N.J., Leonard G.H., Baechler B.R. (2024). Exposure of U.S. adults to microplastics from commonly-consumed proteins. Environ. Pollut..

[45] Othman A.M., Elsayed A.A., Sabry Y.M., Khalil D., Bourouina T. (2023). Detection of Sub-20 μm Microplastic Particles by Attenuated Total Reflection Fourier Transform Infrared Spectroscopy and Comparison with Raman Spectroscopy. ACS Omega.

[46] Asamoah B.O., Uurasjärvi E., Räty J., Koistinen A., Roussey M., Peiponen K.E. (2021). Towards the Development of Portable and In Situ Optical Devices for Detection of Micro-and Nanoplastics in Water: A Review on the Current Status. Polymers.

[47] Cai L., Wang J., Peng J., Tan Z., Zhan Z., Tan X., Chen Q. (2017). Characteristic of microplastics in the atmospheric fallout from Dongguan city, China: Preliminary research and first evidence. Environ. Sci. Pollut. Res..

[48] Dris R., Gasperi J., Saad M., Mirande C., Tassin B. (2016). Synthetic fibers in atmospheric fallout: A source of microplastics in the environment?. Mar. Pollut. Bull..

[49] Sandt C., Waeytens J., Deniset-Besseau A., Nielsen-Leroux C., Réjasse A. (2021). Use and misuse of FTIR spectroscopy for studying the bio-oxidation of plastics. Spectrochim. Acta Part A Mol. Biomol. Spectrosc..

[50] Tang P.L., Forster R., McCumskay R., Rogerson M., Waller C. Handheld FT-IR Spectroscopy for the Triage of Micro- and Meso-Sized Plastics in the Marine Environment Incorporating an Accelerated Weathering Study and an Aging Estimation. Spectroscopy. https://www.spectroscopyonline.com/view/handheld-ft-ir-spectroscopy-triage-micro-and-meso-sized-plastics-marine-environment-incorporating-ac.

[51] Nava V., Frezzotti M.L., Leoni B. (2021). Raman Spectroscopy for the Analysis of Microplastics in Aquatic Systems. Appl. Spectrosc..

[52] Schymanski D., Goldbeck C., Humpf H.-U., Fürst P. (2018). Analysis of microplastics in water by micro-Raman spectroscopy: Release of plastic particles from different packaging into mineral water. Water Res..

[53] PublicSpectra|Raman Spectral Database. https://publicspectra.com/.

[54] Clunies-Ross P.J., Smith G.P.S., Gordon K.C., Gaw S. (2016). Synthetic shorelines in New Zealand? Quantification and characterisation of microplastic pollution on Canterbury’s coastlines. N. Z. J. Mar. Freshw. Res..

[55] Gillibert R., Balakrishnan G., Deshoules Q., Tardivel M., Magazzù A., Donato M.G., Maragò O.M., Lamy De La Chapelle M., Colas F., Lagarde F. (2019). Raman tweezers for small microplastics and nanoplastics identification in seawater. Environ. Sci. Technol..

[56] Ramsperger A.F.R.M., Narayana V.K.B., Gross W., Mohanraj J., Thelakkat M., Greiner A., Schmalz H., Kress H., Laforsch C. (2020). Environmental exposure enhances the internalization of microplastic particles into cells. Sci. Adv..

[57] Dong M., Zhang Q., Xing X., Chen W., She Z., Luo Z. (2020). Raman spectra and surface changes of microplastics weathered under natural environments. Sci. Total Environ..

[58] Analytical Chemistry—A Guide to Proton Nuclear Magnetic Resonance (NMR)–Compound Interest. https://www.compoundchem.com/2015/02/24/proton-nmr/.

[59] Corti A., Vinciguerra V., Iannilli V., Pietrelli L., Manariti A., Bianchi S., Petri A., Cifelli M., Domenici V., Castelvetro V. (2020). Thorough Multianalytical Characterization and Quantification of Micro- and Nanoplastics from Bracciano Lake’s Sediments. Sustainability.

[60] Peez N., Janiska M.-C., Imhof W. (2019). The first application of quantitative 1H NMR spectroscopy as a simple and fast method of identification and quantification of microplastic particles (PE, PET, and PS). Anal. Bioanal. Chem..

[61] Papini G., Petrella G., Cicero D.O., Boglione C., Rakaj A. (2024). Identification and quantification of polystyrene microplastics in marine sediments facing a river mouth through NMR spectroscopy. Mar. Pollut. Bull..

[62] Peez N., Rinesch T., Kolz J., Imhof W. (2022). Applicable and cost-efficient microplastic analysis by quantitative 1 H-NMR spectroscopy using benchtop NMR and NoD methods. Magn. Reson. Chem..

[63] Giannattasio A., Iuliano V., Oliva G., Giaquinto D., Capacchione C., Cuomo M.T., Hasan S.W., Choo K.-H., Korshin G.V., Barceló D. (2024). Micro(nano)plastics from synthetic oligomers persisting in Mediterranean seawater: Comprehensive NMR analysis, concerns and origins. Environ. Int..

[64] Li C., Gao Y., He S., Chi H.-Y., Li Z.-C., Zhou X.-X., Yan B. (2021). Quantification of Nanoplastic Uptake in Cucumber Plants by Pyrolysis Gas Chromatography/Mass Spectrometry. Environ. Sci. Technol. Lett..

[65] Jiménez-Lamana J., Marigliano L., Allouche J., Grassl B., Szpunar J., Reynaud S. (2020). A Novel Strategy for the Detection and Quantification of Nanoplastics by Single Particle Inductively Coupled Plasma Mass Spectrometry (ICP-MS). Anal. Chem..

[66] Bühler C., Simon W. (1970). Curie Point Pyrolysis Gas Chromatography. J. Chromatogr. Sci..

[67] Fischer M., Scholz-Böttcher B.M. (2017). Simultaneous Trace Identification and Quantification of Common Types of Microplastics in Environmental Samples by Pyrolysis-Gas Chromatography–Mass Spectrometry. Environ. Sci. Technol..

[68] Brits M., van Velzen M.J.M., Sefiloglu F.Ö., Scibetta L., Groenewoud Q., Garcia-Vallejo J.J., Vethaak A.D., Brandsma S.H., Lamoree M.H. (2024). Quantitation of micro and nanoplastics in human blood by pyrolysis-gas chromatography–mass spectrometry. Microplast. Nanoplast..

[69] Chen H., Shan X., Qiu X., Ding L., Liang X., Guo X. (2024). High-Resolution Mass Spectrometry Combined with Reactive Oxygen Species Reveals Differences in Photoreactivity of Dissolved Organic Matter from Microplastic Sources in Aqueous Environments. Environ. Sci. Technol..

[70] Arenas-Guerrero P., Delgado Á.V., Donovan K.J., Scott K., Bellini T., Mantegazza F., Jiménez M.L. (2018). Determination of the size distribution of non-spherical nanoparticles by electric birefringence-based methods. Sci. Rep..

[71] Hanif M.A., Ibrahim N., Dahalan F.A., Md Ali U.F., Hasan M., Jalil A.A. (2022). Microplastics and nanoplastics: Recent literature studies and patents on their removal from aqueous environment. Sci. Total Environ..

[72] Kumar A., Dixit C.K., Nimesh S., Chandra R., Gupta N. (2017). 3-Methods for characterization of nanoparticles. Advances in Nanomedicine for the Delivery of Therapeutic Nucleic Acids.

[73] Singh S., Dash A.K., Agrawal S. (2024). Semisolid Dosage Forms. Pharmaceutics.

[74] Misra A., Zambrzycki C., Kloker G., Kotyrba A., Anjass M.H., Franco Castillo I., Mitchell S.G., Güttel R., Streb C. (2020). Water Purification and Microplastics Removal Using Magnetic Polyoxometalate-Supported Ionic Liquid Phases (magPOM-SILPs). Angew. Chemie Int. Ed..

[75] Batool A., Valiyaveettil S. (2021). Surface functionalized cellulose fibers–A renewable adsorbent for removal of plastic nanoparticles from water. J. Hazard. Mater..

[76] Lu Z. (2021). Lovibond^®^ Water Testing. https://www.tpomag.com/uploads/downloads/WHITE_PAPER_TB_SERIES_LOD_V1_EN.pdf.

[77] Sadar M.J. (1995). Stabilized Formazin Composition. https://patents.google.com/patent/US5777011A/en.

[78] Elkhatib D., Oyanedel-Craver V., Carissimi E. (2021). Electrocoagulation applied for the removal of microplastics from wastewater treatment facilities. Sep. Purif. Technol..

[79] Aslan T., Arslan S., Eyvaz M., Güçlü S., Yüksel E., Koyuncu İ. (2016). A novel nanofiber microfiltration membrane: Fabrication and characterization of tubular electrospun nanofiber (TuEN) membrane. J. Memb. Sci..

[80] Rajala K., Grönfors O., Hesampour M., Mikola A. (2020). Removal of microplastics from secondary wastewater treatment plant effluent by coagulation/flocculation with iron, aluminum and polyamine-based chemicals. Water Res..

[81] Malvern Panalytical Basic Principles of Nanoparticle Tracking Analysis–Q&A. Malvern Panalytical. https://www.malvernpanalytical.com/en/learn/knowledge-center/insights/basic-principles-of-nanoparticle-tracking-analysis-qa#:~:text=DLScanmeasuremostmaterials,%2Cand~40nmforliposomes.

[82] Filipe V., Hawe A., Jiskoot W. (2010). Critical Evaluation of Nanoparticle Tracking Analysis (NTA) by NanoSight for the Measurement of Nanoparticles and Protein Aggregates. Pharm. Res..

[83] Lambert S., Wagner M. (2016). Characterisation of nanoplastics during the degradation of polystyrene. Chemosphere.

[84] Meier F., Heinzmann G. (2017). Field-Flow Fractionation: A Powerful Technology for the Separation and Advanced Characterization of Proteins, Antibodies, Viruses, Polymers and Nano-/Microparticles. https://www.researchgate.net/publication/319505751_Field-Flow_Fractionation_A_powerful_technology_for_the_separation_and_advanced_characterization_of_proteins_antibodies_viruses_polymers_and_nano-microparticles.

[85] Williams S.K.R., Runyon J.R., Ashames A.A. (2011). Field-Flow Fractionation: Addressing the Nano Challenge. Anal. Chem..

[86] Ratna D. (2022). Chapter 6-Characterization, Performance Evaluation and Lifetime Analysis of Thermoset Resin.

[87] Tanzi M.C., Farè S., Candiani G., Tanzi M.C., Farè S. (2019). Chapter 7-Techniques of Analysis.

[88] Gill P., Moghadam T.T., Ranjbar B. (2010). Differential scanning calorimetry techniques: Applications in biology and nanoscience. J. Biomol. Tech..

[89] (2015). A Beginner’s Guide.

[90] Wies S., Geyer A., Eysel W. (1992). The limit of detection in differential scanning calorimetry. J. Therm. Anal..

[91] Majewsky M., Bitter H., Eiche E., Horn H. (2016). Determination of microplastic polyethylene (PE) and polypropylene (PP) in environmental samples using thermal analysis (TGA-DSC). Sci. Total Environ..

[92] Bitter H., Lackner S. (2021). Fast and easy quantification of semi-crystalline microplastics in exemplary environmental matrices by differential scanning calorimetry (DSC). Chem. Eng. J..

[93] Procházková P., Kalčíková G., Maršálková E., Zlámalová Gargošová H., Kučerík J. (2024). Innovative approach for quantitative determination of ingested microplastics by *Daphnia magna*: Use of differential scanning calorimetry and thermogravimetry. J. Therm. Anal. Calorim..

[94] Kurzweg L., Hauffe M., Schirrmeister S., Adomat Y., Socher M., Grischek T., Fery A., Harre K. (2024). Microplastic analysis in sediments of the Elbe River by electrostatic separation and differential scanning calorimetry. Sci. Total Environ..

[95] Wlasits P.J., Stoellner A., Lattner G., Maggauer K., Winkler P.M. (2021). Size characterization and detection of aerosolized nanoplastics originating from evaporated thermoplastics. Aerosol Sci. Technol..

[96] Joy D.C., Pawley J.B. (1992). High-resolution scanning electron microscopy. Ultramicroscopy.

[97] Image Resolution.

[98] Mariano S., Tacconi S., Fidaleo M., Rossi M., Dini L. (2021). Micro and Nanoplastics Identification: Classic Methods and Innovative Detection Techniques. Front. Toxicol..

[99] Hoogenboom B.W. (2021). Stretching the resolution limit of atomic force microscopy. Nat. Struct. Mol. Biol..

[100] Yan H., Shirato N., Zhu X., Rosenmann D., Tong X., Xu W., Petrovic C., Rose V., Nazaretski E. (2019). X-ray Assisted Scanning Tunneling Microscopy and Its Applications for Materials Science: The First Results on Cu Doped ZrTe3. Crystals.

[101] Yang T., Luo J., Nowack B. (2021). Characterization of Nanoplastics, Fibrils, and Microplastics Released during Washing and Abrasion of Polyester Textiles. Environ. Sci. Technol..

[102] Shim W.J., Song Y.K., Hong S.H., Jang M. (2016). Identification and quantification of microplastics using Nile Red staining. Mar. Pollut. Bull..

[103] Maes T., Jessop R., Wellner N., Haupt K., Mayes A.G. (2017). A rapid-screening approach to detect and quantify microplastics based on fluorescent tagging with Nile Red. Sci. Rep..

[104] Araujo C.F., Nolasco M.M., Ribeiro A.M.P., Ribeiro-Claro P.J.A. (2018). Identification of microplastics using Raman spectroscopy: Latest developments and future prospects. Water Res..

[105] Valsesia A., Parot J., Ponti J., Mehn D., Marino R., Melillo D., Muramoto S., Verkouteren M., Hackley V.A., Colpo P. (2021). Detection, counting and characterization of nanoplastics in marine bioindicators: A proof of principle study. Microplast. Nanoplast..

[106] Schwaferts C., Niessner R., Elsner M., Ivleva N.P. (2019). Methods for the analysis of submicrometer- and nanoplastic particles in the environment. TrAC Trends Anal. Chem..

[107] Coppock R.L., Cole M., Lindeque P.K., Queirós A.M., Galloway T.S. (2017). A small-scale, portable method for extracting microplastics from marine sediments. Environ. Pollut..

[108] Díaz-Jaramillo M., Islas M.S., Gonzalez M. (2021). Spatial distribution patterns and identification of microplastics on intertidal sediments from urban and semi-natural SW Atlantic estuaries. Environ. Pollut..

[109] Crichton E.M., Noël M., Gies E.A., Ross P.S. (2017). A novel, density-independent and FTIR-compatible approach for the rapid extraction of microplastics from aquatic sediments. Anal. Methods.

[110] Thomas D., Schütze B., Heinze W.M., Steinmetz Z. (2020). Sample Preparation Techniques for the Analysis of Microplastics in Soil—A Review. Sustainability.

[111] Marchetti A., Beltran V., Nuyts G., Borondics F., De Meyer S., Van Bos M., Jaroszewicz J., Otten E., Debulpaep M., De Wael K. (2022). Novel optical photothermal infrared (O-PTIR) spectroscopy for the noninvasive characterization of heritage glass-metal objects. Sci. Adv..

[112] Gvazava N., Konings S.C., Cepeda-Prado E., Skoryk V., Umeano C.H., Dong J., Silva I.A.N., Ottosson D.R., Leigh N.D., Wagner D.E. (2023). Label-Free High-Resolution Photothermal Optical Infrared Spectroscopy for Spatiotemporal Chemical Analysis in Fresh, Hydrated Living Tissues and Embryos. J. Am. Chem. Soc..

[113] Tarafdar A., Xie J., Gowen A., O’Higgins A.C., Xu J.-L. (2024). Advanced optical photothermal infrared spectroscopy for comprehensive characterization of microplastics from intravenous fluid delivery systems. Sci. Total Environ..

[114] Tarafdar A., Choi S.-H., Kwon J.-H. (2022). Differential staining lowers the false positive detection in a novel volumetric measurement technique of microplastics. J. Hazard. Mater..

[115] Bai Y., Zhang B., Xu N., Zhou J., Shi J., Diao Z. (2023). Vision-based navigation and guidance for agricultural autonomous vehicles and robots: A review. Comput. Electron. Agric..

[116] Hilfiker J.N., Hong N., Schoeche S. (2022). Mueller matrix spectroscopic ellipsometry. Adv. Opt. Technol..

[117] Li J., Liu H., Liao R., Wang H., Chen Y., Xiang J., Xu X., Ma H. (2023). Recognition of microplastics suspended in seawater via refractive index by Mueller matrix polarimetry. Mar. Pollut. Bull..

[118] Li J., Wang H., Liao R., Wang Y., Liu Z., Zhuo Z., Guo Z., Ma H. (2021). Statistical Mueller matrix driven discrimination of suspended particles. Opt. Lett..

[119] Lopera M.J., Trusiak M., Doblas A., Ottevaere H., Trujillo C. (2024). Mueller-Gabor holographic microscopy. Opt. Lasers Eng..

[120] Huang Z., Cao L. (2024). Quantitative phase imaging based on holography: Trends and new perspectives. Light Sci. Appl..

[121] Li Y., Zhu Y., Huang J., Ho Y.-W., Fang J.K.-H., Lam E.Y. (2024). High-throughput microplastic assessment using polarization holographic imaging. Sci. Rep..

[122] Valentino M., Sirico D.G., Memmolo P., Miccio L., Bianco V., Ferraro P. (2023). Digital holographic approaches to the detection and characterization of microplastics in water environments. Appl. Opt..

[123] Valentino M., Běhal J., Bianco V., Itri S., Mossotti R., Dalla Fontana G., Battistini T., Stella E., Miccio L., Ferraro P. (2022). Intelligent polarization-sensitive holographic flow-cytometer: Towards specificity in classifying natural and microplastic fibers. Sci. Total Environ..

[124] Zhu Y., Li Y., Huang J., Lam E.Y. (2024). Smart polarization and spectroscopic holography for real-time microplastics identification. Commun. Eng..

[125] Tofa T.S., Kunjali K.L., Paul S., Dutta J. (2019). Visible light photocatalytic degradation of microplastic residues with zinc oxide nanorods. Environ. Chem. Lett..

[126] Sakr O.S., Borchard G. (2013). Encapsulation of Enzymes in Layer-by-Layer (LbL) Structures: Latest Advances and Applications. Biomacromolecules.

[127] Talbert J.N., Goddard J.M. (2013). Influence of nanoparticle diameter on conjugated enzyme activity. Food Bioprod. Process..

[128] Ji Y., Mao G., Wang Y., Bartlam M. (2013). Structural insights into diversity and n-alkane biodegradation mechanisms of alkane hydroxylases. Front. Microbiol..

[129] Austin H.P., Allen M.D., Donohoe B.S., Rorrer N.A., Kearns F.L., Silveira R.L., Pollard B.C., Dominick G., Duman R., El Omari K. (2018). Characterization and engineering of a plastic-degrading aromatic polyesterase. Proc. Natl. Acad. Sci. USA.

[130] Wei R., Zimmermann W. (2017). Microbial enzymes for the recycling of recalcitrant petroleum-based plastics: How far are we?. Microb. Biotechnol..

[131] Sadler J.C., Wallace S. (2021). Microbial synthesis of vanillin from waste poly(ethylene terephthalate). Green Chem..

[132] Choi E.J., Jin H.M., Lee S.H., Math R.K., Madsen E.L., Jeon C.O. (2013). Comparative genomic analysis and benzene, toluene, ethylbenzene, and o-, m-, and p-xylene (BTEX) degradation pathways of *Pseudoxanthomonas spadix* BD-a59. Appl. Environ. Microbiol..

[133] Zhe Z., Peng H., Yang D., Zhang G., Zhang J., Ju F. (2021). Polyvinyl Chloride Biodegradation Fuels Survival of Invasive Insect Larva and Intestinal Degrading Strain of Klebsiella. bioRxiv.

[134] Yasuhira K., Tanaka Y., Shibata H., Kawashima Y., Ohara A., Kato D., Takeo M., Negoro S. (2007). 6-Aminohexanoate Oligomer Hydrolases from the Alkalophilic Bacteria Agromyces sp. Strain KY5R and Kocuria sp. Strain KY2. Appl. Environ. Microbiol..

[135] Matés J.M., Pérez-Gómez C., De Castro I.N. (1999). Antioxidant enzymes and human diseases. Clin. Biochem..

[136] Yu C., Shao Z., Liu B., Zhang Y., Wang S. (2016). Inhibition of 2-Amino-1-methyl-6-phenylimidazo [4,5-b]pyridine (PhIP) Formation by Alkoxy Radical Scavenging of Flavonoids and Their Quantitative Structure–Activity Relationship in a Model System. J. Food Sci..

[137] Arregui L., Ayala M., Gómez-Gil X., Gutiérrez-Soto G., Hernández-Luna C.E., Herrera De Los Santos M., Levin L., Rojo-Domínguez A., Romero-Martínez D., Saparrat M.C.N. (2019). Laccases: Structure, function, and potential application in water bioremediation. Microb. Cell Factories.

[138] Oliveira A.R., Mota C., Mourato C., Domingos R.M., Santos M.F.A., Gesto D., Guigliarelli B., Santos-Silva T., Romão M.J., Cardoso Pereira I.A. (2020). Toward the Mechanistic Understanding of Enzymatic CO_2_ Reduction. ACS Catal..

[139] Metcalf L., Eddy H.P., Tchobanoglous G. (1979). Wastewater Engineering: Treatment, Disposal, Reuse.

